# The spatial relationship between the MRI lesion and intraoperative electrocorticography in focal epilepsy surgery

**DOI:** 10.1093/braincomms/fcac302

**Published:** 2022-11-21

**Authors:** Sven Straumann, Eline Schaft, Herke Jan Noordmans, Jan Willem Dankbaar, Willem M Otte, Josee van Steenis, Paul Smits, Willemiek Zweiphenning, Pieter van Eijsden, Tineke Gebbink, Luigi Mariani, Maryse A van’t Klooster, Maeike Zijlmans

**Affiliations:** Department of Neurology and Neurosurgery, University Medical Center Utrecht, 3584 CX Utrecht, The Netherlands; Department of Neurosurgery, University Hospital Basel, 4051 Basel, Switzerland; Department of Anaesthesiology and Pain Medicine, Inselspital, University Hospital Bern, 3010 Bern, Switzerland; Department of Neurology and Neurosurgery, University Medical Center Utrecht, 3584 CX Utrecht, The Netherlands; Department of Medical Technology and Clinical Physics, University Medical Center Utrecht, 3584 CX Utrecht, The Netherlands; Department of Radiology, University Medical Center Utrecht, 3584 CX Utrecht, The Netherlands; Department of Child Neurology, University Medical Center Utrecht, 3584 CX Utrecht, The Netherlands; Department of Neurology and Neurosurgery, University Medical Center Utrecht, 3584 CX Utrecht, The Netherlands; Faculty of Science and Technology, University of Twente, 7522 NB Enschede, The Netherlands; Department of Neurology and Neurosurgery, University Medical Center Utrecht, 3584 CX Utrecht, The Netherlands; Department of Neurology and Neurosurgery, University Medical Center Utrecht, 3584 CX Utrecht, The Netherlands; Department of Neurology and Neurosurgery, University Medical Center Utrecht, 3584 CX Utrecht, The Netherlands; Department of Neurology and Neurosurgery, University Medical Center Utrecht, 3584 CX Utrecht, The Netherlands; Department of Neurosurgery, University Hospital Basel, 4051 Basel, Switzerland; Department of Neurology and Neurosurgery, University Medical Center Utrecht, 3584 CX Utrecht, The Netherlands; Department of Neurology and Neurosurgery, University Medical Center Utrecht, 3584 CX Utrecht, The Netherlands; Stichting Epilepsie Instellingen Nederland (SEIN), 2103 SW Heemstede, The Netherlands

**Keywords:** epilepsy surgery, electrocorticography, MRI, high-frequency oscillations, spikes

## Abstract

MRI and intraoperative electrocorticography are often used in tandem to delineate epileptogenic tissue in resective surgery for focal epilepsy. Both the resection of the MRI lesion and tissue with high rates of electrographic discharges on electrocorticography, e.g. spikes and high-frequency oscillations (80–500 Hz), lead to a better surgical outcome. How MRI and electrographic markers are related, however, is currently unknown. The aim of this study was to find the spatial relationship between MRI lesions and spikes/high-frequency oscillations. We retrospectively included 33 paediatric and adult patients with lesional neocortical epilepsy who underwent electrocorticography-tailored surgery (14 females, median age = 13.4 years, range = 0.6–47.0 years). Mesiotemporal lesions were excluded. We used univariable linear regression to find correlations between pre-resection spike/high-frequency oscillation rates on an electrode and its distance to the MRI lesion. We tested straight lines to the centre and the edge of the MRI lesion, and the distance along the cortical surface to determine which of these distances best reflects the occurrence of spikes/high-frequency oscillations. We conducted a moderator analysis to investigate the influence of the underlying pathology type and lesion volume on our results. We found spike and high-frequency oscillation rates to be spatially linked to the edge of the MRI lesion. The underlying pathology type influenced the spatial relationship between spike/high-frequency oscillation rates and the MRI lesion (*P*_spikes_ < 0.0001, *P*_ripples_ < 0.0001), while the lesion volume did not (*P*_spikes_ = 0.64, *P*_ripples_ = 0.89). A higher spike rate was associated with a shorter distance to the edge of the lesion for cavernomas [*F*(1,64) = −1.37, *P* < 0.0001, *η*^2^ = 0.22], focal cortical dysplasias [*F*(1,570) = −0.25, *P* < 0.0001, *η*^2^ = 0.05] and pleomorphic xanthoastrocytomas [*F*(1,66) = −0.18, *P* = 0.01, *η*^2^ = 0.09]. In focal cortical dysplasias, a higher ripple rate was associated with a shorter distance [*F*(1,570) = −0.35, *P* < 0.0001, *η*^2^ = 0.05]. Conversely, low-grade gliomas showed a positive correlation; the further an electrode was away from the lesion, the higher the rate of spikes [*F*(1,75) = 0.65, *P* < 0.0001, *η*^2^ = 0.37] and ripples [*F*(1,75) = 2.67, *P* < 0.0001, *η*^2^ = 0.22]. Pathophysiological processes specific to certain pathology types determine the spatial relationship between the MRI lesion and electrocorticography results. In our analyses, non-tumourous lesions (focal cortical dysplasias and cavernomas) seemed to intrinsically generate spikes and high-frequency oscillations, particularly at the border of the lesion. This advocates for a resection of this tissue. Low-grade gliomas caused epileptogenicity in the peritumoural tissue. Whether a resection of this tissue leads to a better outcome is unclear. Our results suggest that the underlying pathology type should be considered when intraoperative electrocorticography is interpreted.

## Teaser

Straumann *et al*. studied the electrical brain activity of patients undergoing epilepsy surgery. They report that certain diseases influence where epileptic signal patterns occur in relation to abnormalities on MRI scans. Their findings could help to make the neurosurgical removal of diseased tissue more precise.

## Graphical Abstract

**Graphical abstract:**
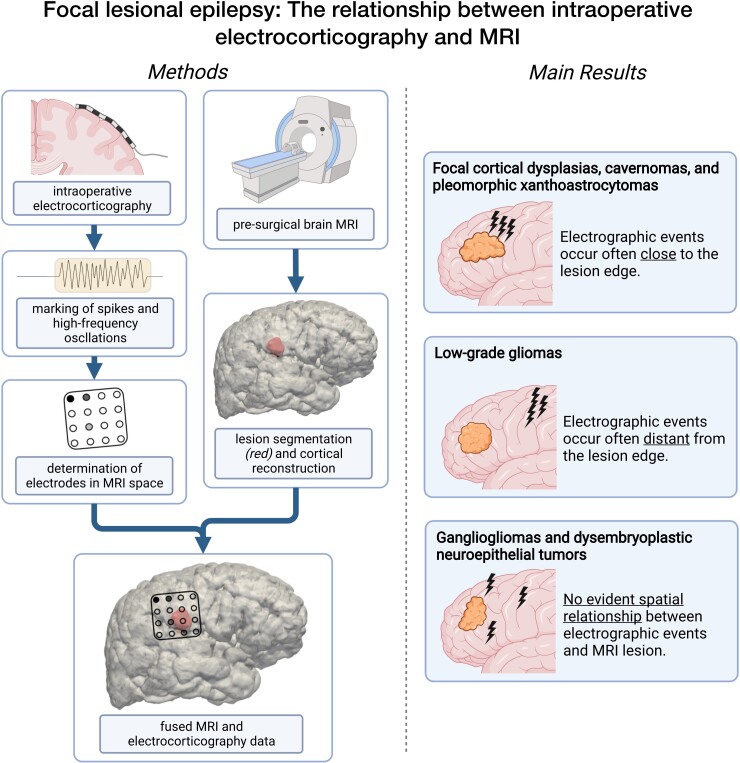

## Introduction

Up to 30–40% of people with focal epilepsy cannot achieve adequate seizure control despite optimized drug treatment.^1–3^ In many of these cases, the surgical resection of the presumed seizure focus is an effective and safe treatment option.^1,3,4^ The accurate localization of epileptogenic tissue is paramount for a good surgical outcome.^1,5–7^ Rosenow and Lüders^8^ defined the minimal theoretical resection area to ensure seizure freedom as the ‘epileptogenic zone’ (EZ). More recent evidence has led to an emerging view of the EZ not as a static area of tissue but an interconnected network within the epileptic brain.^9–14^

During pre-surgical workup, MRI scans form a vital basis for the localization of structural lesions associated with epilepsy.^1,4,15,16^ Patients with a clearly defined MRI lesion are two to three times more likely to achieve postoperative seizure freedom than patients without structural abnormalities on MRI.^1^ But not all epileptogenic pathologies are readily visible on structural MRI scans.^16^ Moreover, even a clearly defined MRI lesion is not necessarily the sole substrate for epileptogenic processes.^9^ Tissue around or at a distance from an MRI lesion may be more epileptogenic than the lesion itself.^17–19^ This implies that MRI lesions, which are often the primary target of surgical resection, may be only part of a broader epileptogenic network.^9^ Additional diagnostic techniques are needed to delineate parts of this network that cannot be detected through MRI alone.

Intraoperative electrocorticography (ioECoG) can assist the localization of the EZ beyond the margins of MRI lesions,^5,7,20–22^ particularly in patients with neocortical lesions.^5,23^ A surgical strategy that combines a complete resection of the MRI lesion and resecting epileptogenic tissue identified through ioECoG has been linked to a better surgical outcome than lesionectomy alone.^5,22^ Limitations of ioECoG include the limited time available to gain accurate recordings during surgery, its dependency on interictal rather than ictal biomarkers and its susceptibility to anaesthesiologic agents.^5,7,20,22^

Epileptiform spikes are the most commonly used ioECoG biomarkers to identify epileptogenic tissue.^8,22^ The cortical area where spikes are found is thought to be larger than the EZ.^5^ Patients often display multiple areas of spike generation, from which spikes are thought to propagate throughout the brain along the epileptogenic network.^10,11,24–26^

In recent decades, high-frequency oscillations (HFOs) have been described as promising electrographic biomarkers, although their clinical application is not yet as widespread as that of spikes.^6,27–29^ HFOs represent prominent, burst-like events with a frequency of >80 Hz. They can be subclassified into ripples (80–250 Hz) and fast ripples (250–500 Hz).^20,27,29^ HFOs, particularly fast ripples,^30,31^ have been shown to be more specific for epileptogenic tissue than spikes.^32,33^ Their complete removal has been linked to a better outcome than the complete removal of spikes.^34,35^ Other studies have put the reliability of HFOs as a biomarker into question.^19,28,36,37^

Structural MRI scans and ioECoG are often used in tandem to localize the EZ. However, no studies have yet investigated the direct relationship between the MRI lesion and electrographic biomarkers recorded on ioECoG. Recent studies indicate that not all areas showing spikes or HFOs may need to be resected for a good surgical outcome.^5,34,36,37^ This can lead to uncertainty because it may not be immediately apparent which areas showing HFOs or spikes on ioECoG need to be resected to improve the surgical outcome and which can be left *in situ* without detriment to the patient. The aim of this study was to better understand the relationship between electrographic markers and the MRI lesion, taking the anatomy and underlying pathology into account.

It is generally assumed that the occurrence of spikes and HFOs is spatially linked to MRI lesions, but to what extent is unclear. We hypothesized that the spike and HFO rates on a given ioECoG electrode correlate with its distance from the MRI lesion. We tested three distances as different models to link ioECoG findings to the MRI lesion. We hypothesized that the distance that best reflects the occurrence of electrographic discharges within the epileptogenic network would correlate strongly with recorded spike and HFO rates. Additionally, we investigated the influence of the underlying pathology type and lesion volume on the relationship between MRI and ioECoG.

## Materials and methods

### Study design and patients

We selected patients from the cohort of participants of the HFO trial, a randomized controlled trial comparing the use of spikes and HFOs for intraoperative neurosurgical guidance.^38^ The results of the HFO trial will be published separately.

Our institute’s medical ethical committee approved the trial protocol and subsequent data analysis. The trial was performed by the principles of the Declaration of Helsinki. All patients and the parents or legal representatives of eligible patients provided written informed consent before enrolment.

We retrospectively included paediatric and adult patients who underwent ioECoG-tailored neurosurgery for focal lesional epilepsy at our institution between October 2014 and January 2020 as a part of the HFO trial that fulfilled the following criteria: (i) Good postsurgical seizure outcome, defined as Class 1a on the Engel Outcome Scale at the 1-year follow-up visit^39^; (ii) availability of a postoperative structural MRIs with no radiographic signs of residual lesional tissue; we excluded (iii) patients who had repeated surgery and (iv) patients with mesiotemporal lesions. We did include neocortical temporal lesions that did not affect the mesiotemporal structures. Patients with non-optimal surgical outcome, incomplete resections and repeated surgeries were excluded because these factors might indicate that the EZ extended further than the area on which ioECoG was performed.

### Intraoperative electrocorticography

#### IoECoG recordings

The surgical approach and placement of ioECoG electrodes were based on the standardized pre-surgical workup at our institution. IoECoG recordings were performed directly on the cortex using standard 4 × 5 or 4 × 4 electrode grids and 1 × 8 electrode strips (Ad-Tech, Racine, WI, USA). Grids and strips consisted of platinum electrodes with a 4.2 mm^2^ contact surface and an interelectrode distance of 1 cm. We used a 64-channel (Micromed LTM Express) or 32-channel (Micromed Flexi) EEG system at a sampling rate of 2048 Hz with an anti-aliasing filter at 538 Hz. The neurosurgical strategy was to remove the MRI lesion based on Brainlab neuronavigation (Brainlab, Munich, Germany) and further intraoperative tailoring of the resection with ioECoG. The intravenous application of propofol was stopped for 5–10 min during each recording until a continuous background pattern was confirmed to minimize the influence of propofol on the ioECoG recordings.^40^ Patients remained unconscious throughout the entire surgical procedure. The ioECoG was reviewed intraoperatively for spikes or HFOs in Stellate Harmonie Viewer (Natus Medical Inc., Montreal, QC, Canada) to guide the resection. Intraoperative photographs of the ioECoG grid positions on the cortex were taken.

#### IoECoG data selection and detection of biomarkers

We included all ioECoG recordings that were taken prior to the start of the resection. IoECoG recordings were retrospectively visually assessed using Brain Quick Software (Micromed SpA). Artefactual channels were excluded. One minute epochs near the end of each recording were selected to detect spikes and HFOs.

Spikes and fast ripples were visually marked by the main author (S.S.) in a bipolar montage. We used conventional settings to identify spikes (1.6–80.0 Hz infinite impulse response filter filter, 500–900 μV/cm gain, 5–10 s/page) and fast ripples [250–1000 Hz finite impulse response (FIR) filter, 10–30 μV/cm gain, 1 s/page].

Ripples were automatically detected in a bipolar montage using a MATLAB-based detector adapted to our data.^41–44^ All 1 min epochs were subsequently visually checked for missing and false positive ripples by the main author (S.S.) using conventional settings (80–1000 Hz FIR filter, 40–60 μV/cm gain, 1 s/page).

All spike, ripple and fast ripple markings were independently verified by two additional researchers (M.Z. and E.S.).

### Pathology type

The underlying pathology type of all lesions was determined using intraoperatively obtained tissue samples as part of routine histopathological analysis after surgery.

### Magnetic resonance imaging

#### MRI acquisition

All patients underwent structural 1.5 or 3T MRI brain scans using a dedicated epilepsy protocol pre- and postoperatively [including a 3D T_1_-weighted and either a T_2_-weighted or fluid attenuated inversion recovery (FLAIR) image].

#### MRI lesion segmentation

We segmented the MRI lesions as binary volumes using the Active Contour Segmentation Mode in ITK-SNAP (Yushkevich *et al*.,^45^www.itksnap.org). Different brain and lesion tissue types were manually marked in the *Classification* pre-segmentation mode based on differences in signal intensities in T_1_-, T_2_- and FLAIR-weighted scans, followed by a semi-automated segmentation of the MRI lesion.

All segmentations were visually verified (and manually corrected, if needed) by the main author (S.S.) and a board-certified neuroradiologist (J.W.D.). Lesion volumes were calculated in *ITK-SNAP* based on the MRI lesion segmentations. Illustrative cases of the lesion segmentation for all pathology types can be found in Supplementary File 1.

### IoECoG electrode coordinates in MRI space

We performed an automatic whole-brain segmentation of the original MRI in FreeSurfer (https://freesurfer.net^46^). This segmentation was then imported into the custom written image analysis software Java Navigation (release 2021),^47^ developed by HK and adapted for this study.

**Figure 1:**
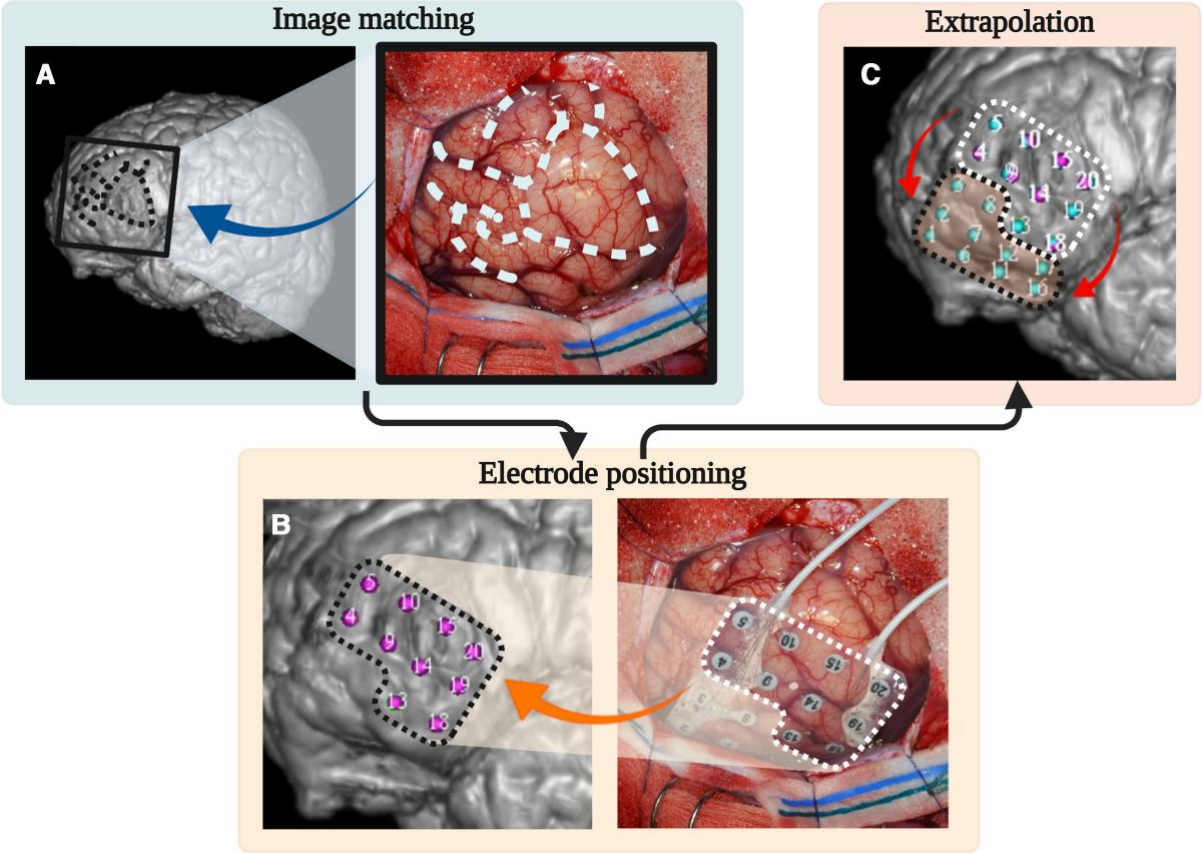
**The determination of ioECoG electrode coordinates in MRI space.** An ioECoG recording of the left frontal lobe is used as an example (Patient 23). (**A**) Intraoperative photographs were visually matched to a cortical reconstruction in Java Navigation using prominent anatomical features (such as gyri, sulci and blood vessels). Dashed lines visualise prominent sulcal patterns used in this case. (**B**) Markers were manually placed for visible electrodes. Dotted lines frame the group of electrodes that are added in this step. (**C**) In an automated step, the positions of all remaining electrodes were extrapolated taking the fixed between-electrode distance of 1 cm and the curvature of the cortical surface into account. Dotted lines frame the electrodes that are added in this step.

We used prominent anatomical features (such as sulci, gyri and blood vessels) to visually match photographs taken during surgery to the cortical reconstruction in Java Navigation. We then manually placed markers for ioECoG electrodes visible on the photographs onto the cortex. An automated extrapolation process, which took the fixed between-electrode distance of 1 cm and the curvature of the cortical surface into account, was used to arrive at the position of any electrodes that were hidden under the skull. This allowed us to obtain coordinates in MRI space for all ioECoG electrodes. For an illustration of this process, see Fig. 1.

### Distances

#### Distances between ioECoG electrodes and MRI lesions

Lesion segmentations, whole-brain segmentations and ioECoG electrode coordinates were imported into MATLAB (The MathWorks Inc., Natick, MA, USA). The bipolar montage used to mark spikes and HFOs was recreated by defining the exact spatial midpoint between two neighbouring electrode coordinates as the bipolar electrode coordinate. We then calculated the following distances:

1. The Euclidian (i.e. *shortest possible*) distance between each bipolar electrode and the closest edge of the lesion.
2. The Euclidian distance between each bipolar electrode and the centre of mass (COM) of the lesion.
3. The geodesic distance between each bipolar electrode and the COM of the lesion, consisting of the shortest possible straight line from the COM to the cortical surface and the shortest line from that point along the cortical surface to the electrode.

**Figure 2:**
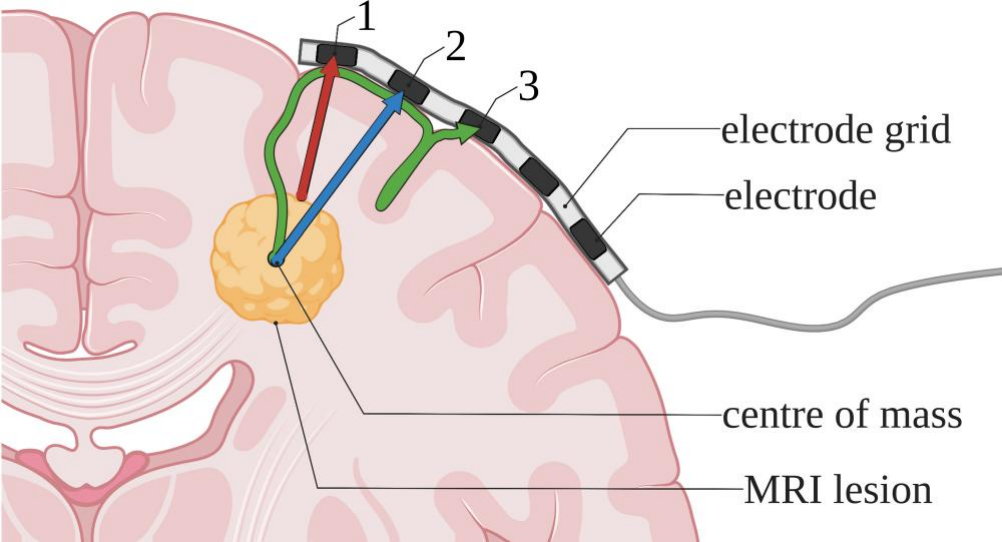
**An illustration of the three different distances between the MRI lesion and ioECoG electrodes.** (1) The Euclidian distance to the closest edge of the lesion (red arrow). (2) The Euclidian distance to the COM of the lesion (blue arrow). (3) The geodesic distance to the COM of the lesion along the cortical surface (green arrow).

The geodesic distance was calculated using the Image Processing Toolbox and the open-source MatImage Library (Legland^48^). For an illustration of the three distances, see Fig. 2. We then determined which distance showed the strongest correlations with ioECoG biomarker rates. We used this distance for additional analyses.

#### Distance to the electrode with most events

We determined the electrode with the highest spike, ripple and fast ripple rate for each patient. The distance to this electrode is called *D*_max_ (spikes), *D*_max_ (ripples) and *D*_max_ (fast ripples), respectively.

### Statistical analysis

We performed all statistical analyses in IBM SPSS Statistics 20 (IBM Corp., Armonk, NY, USA).

We calculated means and standard deviations (SDs) if data were normally distributed and medians and ranges if data were not normally distributed.

We used heteroscedasticity consistent standard errors (using an HC3 Davidson–MacKinnon estimator) in all of our regression models and moderator analyses. For this reason, we report *η*^2^ as the effect size, true to the SPSS output. It should be noted that, in our models, *η*^2^ is numerically equivalent to *r*^2^.^49^ To our knowledge, no results of comparable analyses have been published to date to serve as comparison for the effect sizes of our models. Therefore, we used Cohen’s^50^ original classification for effect sizes: *η*^2^ ≤ 0.06 as *small*, *η*^2^ ≤ 0.14 as *moderate* and *η*^2^ ≥ 0.14 as *large*.

A *P*-value of ≤0.05 was considered statistically significant. We did not correct for multiple comparisons as we tested complementary hypotheses with interdependent variables.

#### The correlation between spike/HFO rates and distance

We used separate univariable linear regression to determine the relationship between the three distances (*dependent variable*) and spike, ripple and fast ripple rates (*independent variable*).

Linear regression was performed for the entire population and for the subgroups based on the underlying pathology. Bootstrapping based on 1000 samples was performed for all linear regression models.

Based on the effect sizes of these linear regressions, we determined which of the three distances illustrated in Fig. 2 best reflects the spatial link between the MRI lesion and ioECoG findings. The distance which showed the strongest correlations to spike/HFO rates was assumed to most likely represent the actual generation of electrographic discharges. This distance was used for all additional analyses.

For fast ripples, we additionally calculated a binary logistical regression with *occurrence* and *non-occurrence* of fast ripples on a channel as a dichotomous dependent variable (independent of the rate of fast ripples). The distance to the lesion was used as the independent variable in this analysis to allow for a binary logistical model.

#### Visual slope comparison

We used a visual slope comparison to test whether our linear regression results align with tendencies visible using only a single data point per patient. We plotted the highest spike and ripple rate for every patient against the distance to the electrode on which it was recorded [*D*_max_ (spikes)/*D*_max_ (ripples)]. We then visually compared the resulting slopes (negative versus positive) per pathology with those of our linear regression models.

#### Comparison of distances to the electrode with most events

We compared the mean *D*_max_ (spikes), *D*_max_ (ripples) and *D*_max_ (fast ripples) using a one-way ANOVA.

#### Moderator analysis of pathology type and lesion volume

We hypothesized that the underlying pathology type and/or lesion volume might influence the spatial relationship between the MRI lesion and ioECoG results. To test this, we conducted a simple slope moderator analysis using the PROCESS macro (version 4) in SPSS developed by Hayes.^51^*Pathology type* was tested as a categorical moderator and *lesion volume* as a continuous moderator.

## Results

### Patients and descriptive statistics

We included 33 patients (14 females, median age = 13.4 years, range = 0.6–47.0 years). Focal cortical dysplasias (FCDs) and gangliogliomas were the most frequent pathologies. Baseline characteristics and mean lesion volumes are outlined in Table 1.

**Table 1:** Patient characteristics

*Sex (number of patients)*		
Male/female		19/14
*Age at surgery (years)*		
Median (range)		13.4 (0.6–47.0)
*Underlying pathology*	*Number of patients (%)*	*Mean volume (SD) (cm^3^)*
FCD	12 (36%)	2.5 (3.0)
Of which Subtype 2a	1	2.1
Of which Subtype 2b	8	3.1 (3.4)
Of which subtype not conclusively identifiable	3	1.0 (0.6)
Ganglioglioma	9 (27%)	10.6 (9.7)
DNET	5 (15%)	12.6 (9.4)
Low-grade glioma (WHO Grade I)	3 (9%)	20.8 (28.6)
PXA	2 (6%)	7.0 (8.2)
Cavernoma	2 (6%)	2.1 (0.01)
*Anatomical location of surgery (number of patients, %)*		
Temporal		18 (55%)
Frontal		11 (33%)
Frontal and temporal		1 (3%)
Central		2 (6%)
Parietal		1 (3%)

Baseline patient characteristics, mean lesion volumes (cm^3^) and anatomical location of surgery.

Between one and five pre-resection ioECoG recordings were performed for each patient, resulting in the initial inclusion of 1578 bipolar ioECoG electrodes. Two 1 × 8 strips had to be excluded because no photograph of their placement was available to reconstruct their coordinates in MRI space. We further excluded 168 bipolar ioECoG electrodes due to artefacts, which left a total of 1396 electrodes for our analyses.

We marked a total of 3519 spikes, 1975 ripples and 181 fast ripples. The mean distance of all bipolar electrodes to the edge of the lesion was 18.9 mm (SD 13.9 mm), electrodes on which spikes were found had a mean distance of 17.9 mm (SD 12.5 mm), electrodes with ripples 18.7 mm (SD 12.2 mm) and with fast ripples 15.6 mm (SD 12.0 mm). Additional descriptive statistics are summarized in Table 2.

**Table 2:** Descriptive statistics on ioECoG biomarkers and distances

IoECoG characteristics	Number of included bipolar channels	Total spikes (mean spikes/min, SD)^a^	Total ripples (mean ripples/min, SD)^a^	Total fast ripples (mean fast ripples/min, SD)^a^
All patients	1396 (100%)	3519 (8.3, SD 11.2)	1975 (5.7, SD 7.7)	181 (3.7, SD 7.0)
FCD	572 (41%)	1870 (9.4, SD 12.8)	1224 (6.0, SD 7.8)	154 (4.4, SD 8.2)
Ganglioglioma	368 (26%)	884 (8.7, SD 10.1)	521 (6.6, SD 7.3)	22 (2.0, SD 1.7)
DNET	245 (18%)	230 (3.7, SD 3.3)	125 (3.7, SD 4.7)	*—* ^b^
Low-grade glioma	77 (6%)	299 (10.0, SD 11.9)	35 (3.9, SD 3.8)	*—* ^b^
PXA	68 (5%)	167 (13.9, SD 15.3)	54 (4.2, SD 4.4)	2 (1.0, SD 0.0)
Cavernoma	66 (5%)	69 (4.1, SD 4.9)	16 (2.3, SD 1.7)	*—* ^b^

Distance characteristics^c^ (mm)	Mean distance to all bipolar electrodes, SD	Mean distance to electrodes with spikes, SD	Mean distance to electrodes with ripples, SD	Mean distance to electrodes with fast ripples, SD
All patients	18.9, SD 13.9	17.9, SD 12.5	18.7, SD 12.2	15.6, SD 12.0
FCD	20.0, SD 13.6	16.8, SD 12.1	17.9, SD 12.8	15.5, SD 13.2
Ganglioglioma	20.4, SD 12.6	20.0, SD 11.0	21.4, SD 11.8	15.4, SD 9.3
DNET	14.3, SD 14.9	11.9, SD 9.9	15.6, SD 9.1	*—* ^b^
Low-grade glioma	15.6, SD 14.6	20.0, SD 16.0	28.6, SD 11.5	*—* ^b^
PXA	17.5, SD 12.4	13.0, SD 7.4	17.1, SD 8.3	15.5, SD 6.3
Cavernoma	26.8, SD 15.0	23.4, SD 16.4	18.1, SD 12.8	*—* ^b^

Total number and rates of ioECoG biomarkers and distance characteristics.

^a^ Mean spike, ripple and fast ripple rates were calculated for channels on which at least one of the respective biomarkers was recorded.

^b^ No fast ripples were recorded for these pathologies.

^c^ Euclidean distance to the edge of the lesion.

### Evaluation of the three distances

#### Entire cohort

The distance to the edge of the lesion showed a negative correlation with spike rates [*F*(1,1394) = −0.10, *P* = 0.03, *η*^2^ = 0.004] and ripple rates [*F*(1,1394) = −0.14, *P* = 0.04, *η*^2^ = 0.003]. The distance to the centre of the lesion showed a negative correlation only with ripple rates [*F*(1,1394) = −0.22, *P* = 0.003, *η*^2^ = 0.01]. The geodesic distance along the cortical surface showed no correlations with ioECoG biomarker rates. None of the three distances showed a correlation with fast ripple rates.

#### Stratified by pathology type

The distance to the edge of the lesion showed a correlation with spike rates for cavernoma, FCDs, low-grade glioma and pleomorphic xanthoastrocytomas (PXAs). Ripple rates correlated for FCDs and low-grade gliomas. Fast ripple rates correlated for gangliogliomas. The results of these linear regressions are discussed in more detail below.

The distance to the centre of the lesion showed correlations for the same pathology types as the distance to the edge, except that it did not correlate with spike rates for PXAs. Effect sizes were either similar for the distance to the centre and the edge of the lesion, or larger for the distance to the edge.

The geodesic distance showed only few correlations: spike rates correlated for dysembryoplastic neuroepithelial tumours (DNETs) and PXAs, and fast ripple rates correlated for PXAs.

In summary, the distance to the edge of the lesion showed the strongest correlations to ioECoG biomarker rates. Hence, this distance was used for all following, additional analyses and figures. All linear regression results are given in Supplementary Table 1.

### Correlations between spike/HFO rates and distance

#### Spike rates

**Figure 3:**
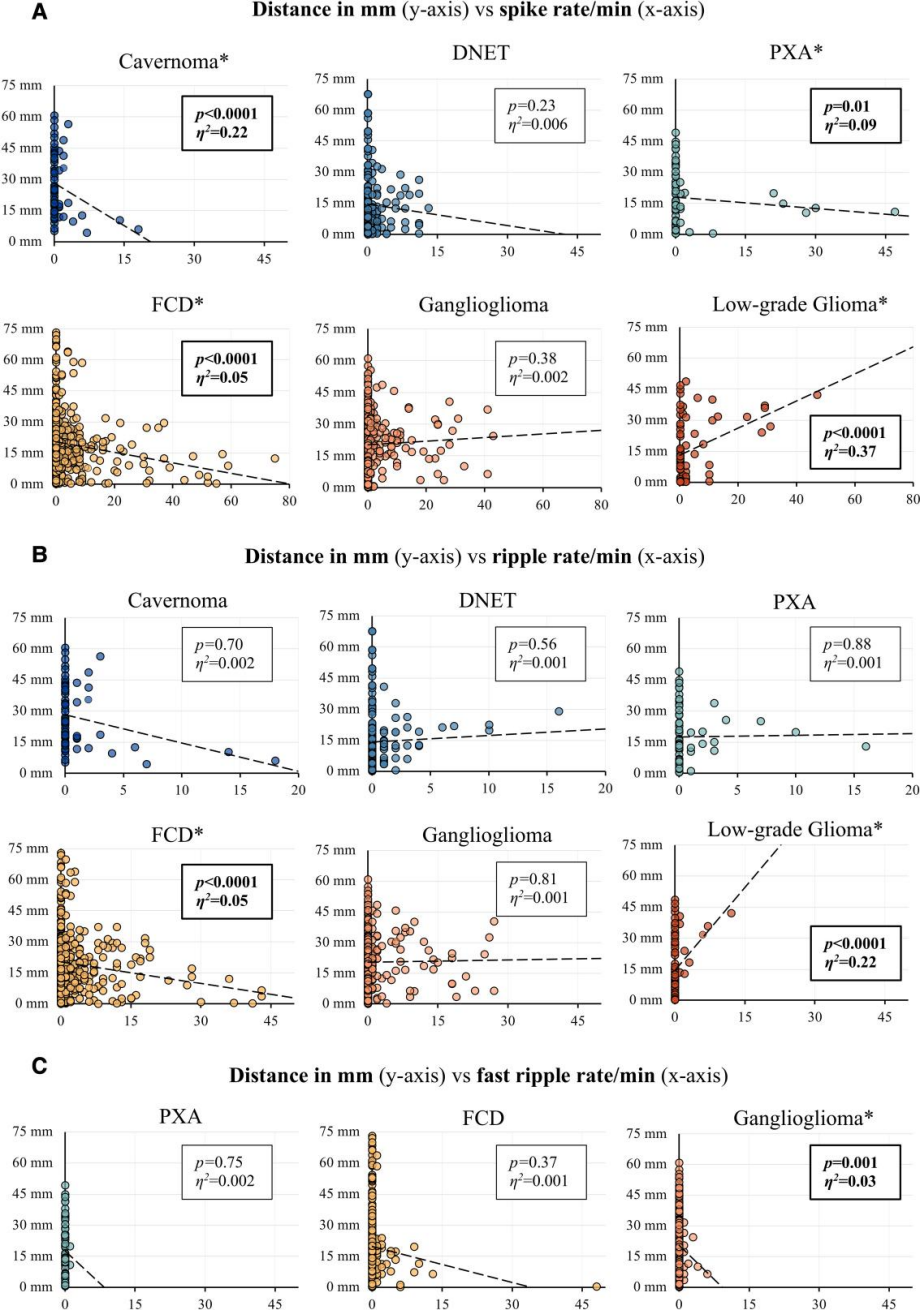
**Scatterplots of distance against spike, ripple and fast ripple rates with linear regression lines.** (**A**) Spike rates, (**B**) ripple rates and (**C**) fast ripple rates per minute (*x-*axis) are plotted against the distance to the edge of the lesion in mm (*y-*axis). Linear regression lines and their respective *P*-value and effect size *η*^2^ are provided. Significant correlations are marked in bold and with an asterisk (*). It should be noted that the scale of the *x*-axis differs from the first to the second row of plots in (**A**) and (**B**). No fast ripples were recorded for cavernomas, DNETs and low-grade gliomas.

A higher spike rate was associated with a shorter distance to the lesion for cavernomas [*F*(1,64) = −1.37, *P* < 0.0001], FCDs [*F*(1,570) = −0.25, *P* < 0.0001] and PXAs [*F*(1,66) = −0.18, *P* = 0.01]. The effect sizes for these negative correlations were: large for cavernomas (*η*^2^ = 0.22), small for FCDs (*η*^2^ = 0.05) and moderate for PXAs (*η*^2^ = 0.09). Conversely, we found a positive correlation between spike rate and distance for low-grade gliomas [*F*(1,75) = 0.65, *P* < 0.0001]. The effect size of this correlation was large (*η*^2^ = 0.37). Spike rates did not correlate with distance for DNETs and gangliogliomas (Fig. 3A).

#### HFO rates

We found correlations for two pathologies between ripple rates and distance: FCDs showed a negative correlation [*F*(1,570) = −0.35, *P* < 0.0001] with a small effect size (*η*^2^ = 0.05), and low-grade gliomas showed a positive correlation [*F*(1,75) = 2.67, *P* < 0.0001] with a large effect size (*η*^2^ = 0.22). Ripple rates did not correlate with distance for cavernomas, DNETs, gangliogliomas and PXAs (Fig. 3B).

No fast ripples were recorded for cavernomas, DNETs and low-grade gliomas. Gangliogliomas showed a negative correlation between fast ripple rates and distance [*F*(1,366) = −2.22, *P* = 0.001] with a small effect size (*η*^2^ = 0.03). No correlation was found for FCDs and PXAs (Fig. 3C).

To test whether the distance to the lesion influenced the *occurrence*, rather than the *rate* of fast ripples, we calculated an additional binary logistic regression for fast ripples. We calculated odds ratios (ORs) for the occurrence of fast ripples for an increase in distance of 1 mm away from the lesion edge. We found no correlation between the occurrence of fast ripples and the distance to the lesion overall [OR, 95% confidence interval (95% CI) = 0.98 (0.96–1.00), *Wald* = 2.94, d.f. = 1, *P* = 0.09]. We also did not find a correlation using a binary logistic model split by pathology types. FCDs showed a tendency for fast ripples to occur close to the MRI lesion; FCD [OR (95% CI) = 0.97 (0.92–1.00), *Wald* = 3.39, d.f. = 1, *P* = 0.06], DNET [OR (95% CI) = 1.02 (0.92–1.13), *Wald* = 0.14, d.f. = 1, *P* = 0.71], ganglioglioma [OR (95% CI) = 0.96 (0.91–1.01), *Wald* = 1.81, d.f. = 1, *P* = 0.18], PXA [OR (95% CI) = 0.99 (0.87–1.11), *Wald* = 0.06, d.f. = 1, *P* = 0.81].

### Distance to the electrode with the maximum event rate

#### Visual slope comparison

**Figure 4:**
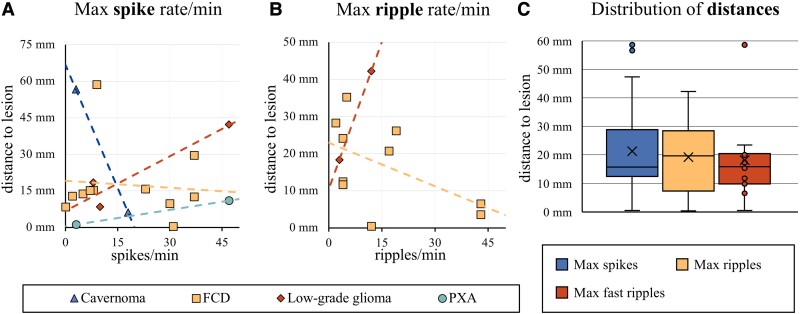
**Distances to electrodes with the highest event rates.** Scatterplots of the maximum recorded (**A**) spike rate in spikes/min and (**B**) ripple rate in ripples/min (*x*-axis) and the distance of that electrode to the lesion edge in mm (*y*-axis). Every patient is represented by a single dot. The slope of the resulting linear trendline (positive versus negative) was visually compared with the linear regression results in Fig. 3. Except for the result for PXAs, all slopes matched those of our earlier results. Except for this visual comparison, no statistical analyses were performed on this data. (**C**) Boxplots of the distribution of the *D*_max_ (spikes; *n* = 18), *D*_max_ (ripples; *n* = 13) and *D*_max_ (fast ripples; *n* = 7). The × marker represents the mean distance. Single data points are shown for *n* < 10. A one-way Welch’s ANOVA revealed no significant differences between the mean distances of spikes, ripples and fast ripples [*F*(6,2283) = 0.98, *P* = 0.41].

All correlations we found through linear regression were consistent with the slopes in this visual analysis, except for PXAs. We found a positive slope for PXAs with single data points per patient, while our linear regression results indicated a negative correlation between spike rate and distance for PXAs (Fig. 4A and B).

#### Mean distances

A one-way Welch’s ANOVA revealed no significant differences between the mean distance to the electrode with the highest rate of spikes, ripples and fast ripples [*F*(6,2283) = 0.98, *P* = 0.41; Fig. 4C].

### The influence of pathology type and lesion volume

#### Mean lesion volumes

**Figure 5:**
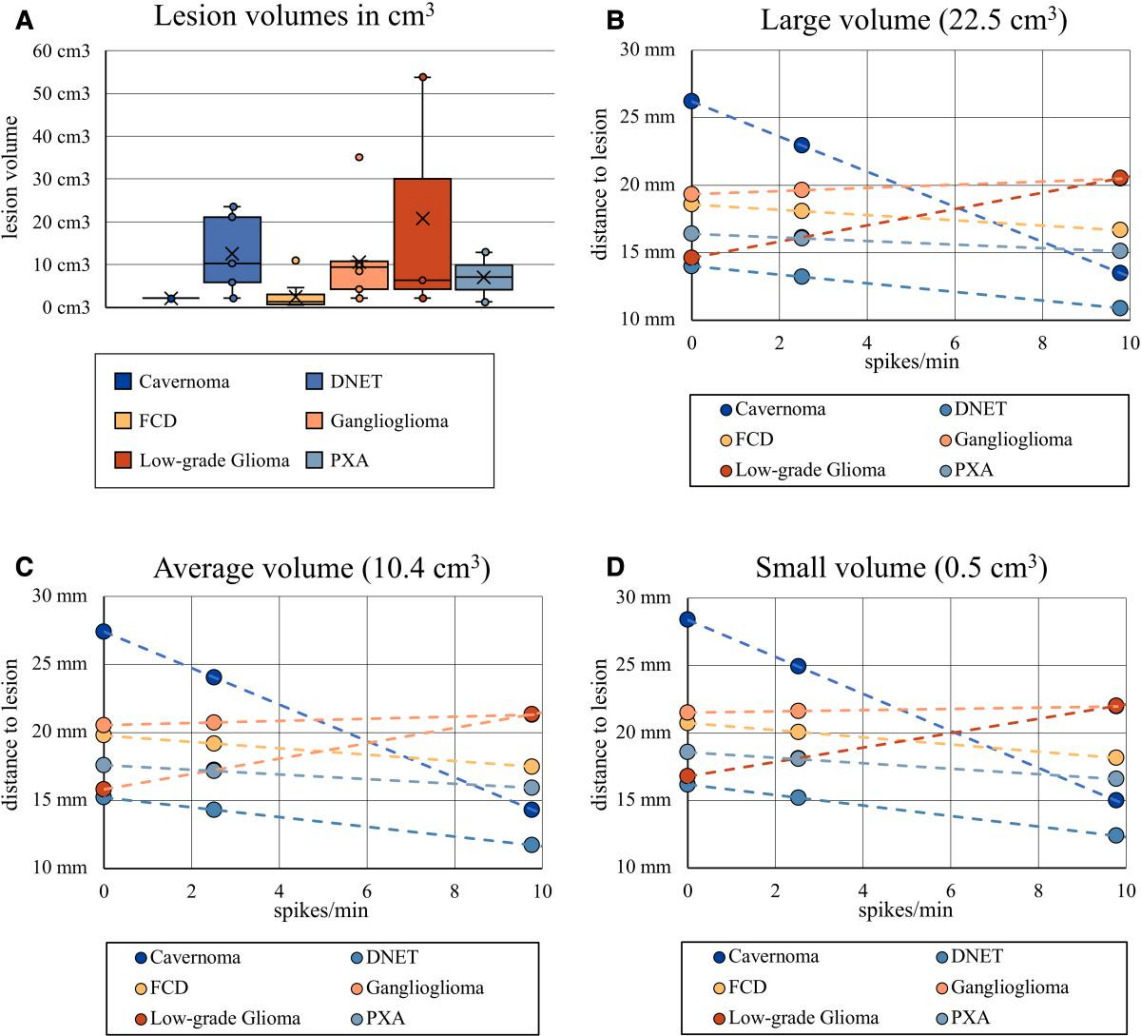
**Illustration of the simple slopes moderator analysis.** (**A**) Boxplots of the lesion volumes stratified by pathology types (*n*_cavernoma_ = 2, *n*_DNET_ = 5, *n*_FCD_ = 12, *n*_ganglioglioma_ = 9, *n*_LG glioma_ = 3, *n*_PXA_ = 2). Single data points are shown for *n* < 10. The × marker represents the mean lesion volume. FCDs and cavernomas tended to have the smallest, low-grade gliomas and the largest lesion volumes. A one-way ANOVA was calculated, which showed that this tendency was not statistically significant [*F*(5,27) = 2.019, *P* = 0.108]. (**B–D**) An illustration of the influence of *pathology type* and *lesion volume* on the relationship between spike rates and distance. (**B–D**) Scatterplots of the mean distance to the edge of the lesion in mm (*y-*axis) against the mean spike rate per minute at that distance (*x-*axis). Regression lines are provided for different pathology types (coloured slopes). Different lesion volumes are represented in the three scatterplots: C = mean lesion volume (10.4 cm^3^); B = large lesion volume (mean + 1 SD = 22.5 cm^3^); D = small lesion volume (mean − 1 SD = 0.5 cm^3^). The regression slopes differ markedly between pathology types within the three scatterplots (**B–D**). However, slopes of the same pathology type vary only insubstantially when compared at large (**B**), average (**C**) and small lesion volumes (**D**). This illustrates the fact that pathology type has a significant influence on the relationship between spike rates and distance [*F*(5,1382) = 14.98, *P* < 0.0001], while lesion volume does not [*F*(1,1382) = 0.21, *P* = 0.64]. It should be noted that the *small*, *average* and *large* volume categories are only exemplary for this illustration. Lesion volume was treated as a continuous moderator in our analysis.

Gliomas (20.8 cm^3^, SD = 28.6 cm^3^) tended to have the largest lesion volumes. FCDs (2.5 cm^3^, SD = 3.0 cm^3^) and cavernomas (2.1 cm^3^, SD = 0.01 cm^3^) tended towards relatively small lesion volumes on MRI (Fig. 5A).

#### Moderator analysis of pathology type and lesion volume

A moderator analysis revealed the underlying pathology type to have a significant effect on the correlation between spike rates and distance overall [*F*(5,1382) = 14.98, *P* < 0.0001] while lesion volume did not [*F*(1,1382) = 0.21, *P* = 0.64; Fig. 5B–D]. Split by pathology types, we found *cavernomas* and *FCDs* to shift the linear regression results towards a more negative slope and *low-grade gliomas* towards a more positive slope. The remaining pathology types did not have a significant moderation effect.

We also found the pathology type to influence the relationship between ripple rates and distance overall [*F*(5,1382) = 8.65, *P* < 0.0001], while lesion volume did not [*F*(1,1382) = 0.02, *P* = 0.89]. This effect was due to low-grade gliomas shifting the linear regression results towards a more positive slope. Other pathology types did not have a significant moderation effect.

Neither pathology type [*F*(2,1000) = 0.07, *P* = 0.93] nor lesion volume [*F*(1,1000) = 0.22, *P* = 0.64] influenced the relationship between fast ripple rates and distance.

All detailed parameters for our moderator analysis can be found in Supplementary Table 2.

## Discussion

We found spike and HFO rates to be spatially linked to the edge of the MRI lesion in patients with focal epilepsy. The nature of this link depended on the underlying pathology type. High spike rates correlated with a shorter distance to the edge of the lesion for cavernomas, FCDs and PXAs. High ripple rates also correlated with a shorter distance for FCDs. Conversely, both high spike and high ripple rates correlated with a longer distance to the lesion for low-grade gliomas.

### The spatial link between spike/HFO rates and the edge of the MRI lesion

We analysed three distances to reflect different possible pathways through which the MRI lesion might trigger epileptogenicity. Two Euclidian distances were chosen to reflect direct recruitment of surrounding tissue by either the centre or the edge of the lesion. The geodesic distance along the cortical surface was chosen to approximate recruitment along horizontal cortico-cortical connections in the absence of actual tractography, which was not part of the routine diagnostic workup for the patients included in this study.

The geodesic distance showed a weaker correlation with spike and HFO rates than the two Euclidian distances. This suggests that a structural lesion most likely causes high spike and HFO rates radially in all directions. This is in line with earlier findings that epileptiform ECoG patterns recruit different areas of non-continuous neocortex at the same time, suggesting subcortical rather than purely cortico-cortical pathways of recruitment.^52,53^ The Euclidian distance to the edge of the lesion was more strongly correlated with spike and HFO rates than the distance to the centre of the lesion. This indicates that spike/HFO occurrence is most likely spatially linked to the lesion edge, which has also been proposed in earlier studies.^54^

In FCDs, electrodes close to the edge of the lesion showed a higher rate of spikes and ripples than those further away from the lesion. This is consistent with earlier findings suggesting the EZ is often congruent with the extent of the lesion in FCDs.^55–57^ It is also in line with the fact that FCDs, particularly Type 2, likely generate epileptic activity intrinsically.^18,57–59^ We found the opposite correlation for low-grade gliomas; the further an electrode was away from the lesion, the higher the rate of spikes and ripples it recorded. This is consistent with earlier findings suggesting that epileptic activity may not be intrinsically generated inside low-grade gliomas but preferentially arises in surrounding peritumoural tissue.^60–62^ The epileptogenicity of tissue surrounding low-grade gliomas may be due to infiltration processes by glioma cells,^60,61^ although neurotransmitter and receptor imbalances, blood–brain barrier disruption, acidosis, hypoxia and inflammation have also been proposed as possible pathomechanisms.^62,63^

Previous studies attribute ambivalent properties to other low-grade tumours in generating epileptic activity. Similar to low-grade gliomas, they can also generate epileptic activity at a distance from the lesion itself,^64^ possibly due to similar invasion processes of the peritumoural tissue.^60^ On the other hand, low-grade tumours like DNETs and gangliogliomas have also been shown to generate seizure activity intrinsically.^62^ This suggests these tumour types may generate epileptic activity both in- and outside the structural lesion. This may explain why we found neither a conclusive positive nor negative correlation between HFO/spike rates and the distance to the radiographic lesion for DNETs and gangliogliomas, while low-grade gliomas showed a positive and FCDs a negative correlation.

Low-grade tumours (low-grade gliomas, DNETs, gangliogliomas and PXAs) tended to have larger lesion volumes than non-tumourous lesions (FCDs and cavernomas). The results of our moderator analysis, however, indicate that lesion volume is not an independent moderator of the spatial relationship between the MRI lesion and spike/HFO rates. Our results suggest that pathophysiological processes specific to the underlying pathology type most likely determine this relationship, independent of the size of the lesion.

Overall, spike rates showed a stronger correlation with the distance to the lesion than HFO rates. This implies that the generation of HFOs is less strongly spatially linked to the MRI lesion than the generation of spikes. This is consistent with the fact that HFOs are often incongruent with radiographic lesional areas and instead correlate with areas involved in seizure generation.^18,65^

The effect sizes of the linear regression results tended to be smaller for pathology types with a relatively large number of included patients, such as FCDs. This may indicate that a large variability between patients exists for the distance to the lesion and the correlating spike/HFO rate. This may have caused effect sizes to get smaller with more included patients per group.

We compared the linear regression models for pathology types that yielded statistically significant results with slopes based on single data points per patient. The type of slope (positive versus negative) was consistent with linear regression results for FCDs, cavernomas and low-grade gliomas. In other words, considering only the electrode with the highest epileptogenicity per patient as opposed to all included electrodes did not change the type of correlation we found. However, the correlation type was inconsistent for PXAs. We believe the linear regression results for PXAs should therefore be interpreted with caution, especially because it is the pathology group with the fewest patients (*n* = 2). Even though the visual slope analyses supported our linear regression results, care should also be taken when interpreting the results for other pathology types with relatively few included patients, such as cavernoma (*n* = 2), low-grade glioma (*n* = 3) and possibly DNET (*n* = 5).

### Clinical relevance of our findings

We found a striking difference between different pathology types in terms of correlation between spike/HFO rates and distance. This indicates that a simple, one-size-fits-all solution may not exist for the intraoperative tailoring of epilepsy surgery using ioECoG. Pathomechanisms specific to certain pathology types likely influence the spatial relationship between the MRI lesion and spike/HFO generation. This suggests that high spike or HFO rates on ioECoG may not warrant the same surgical action for different underlying pathologies.

**Figure 6:**
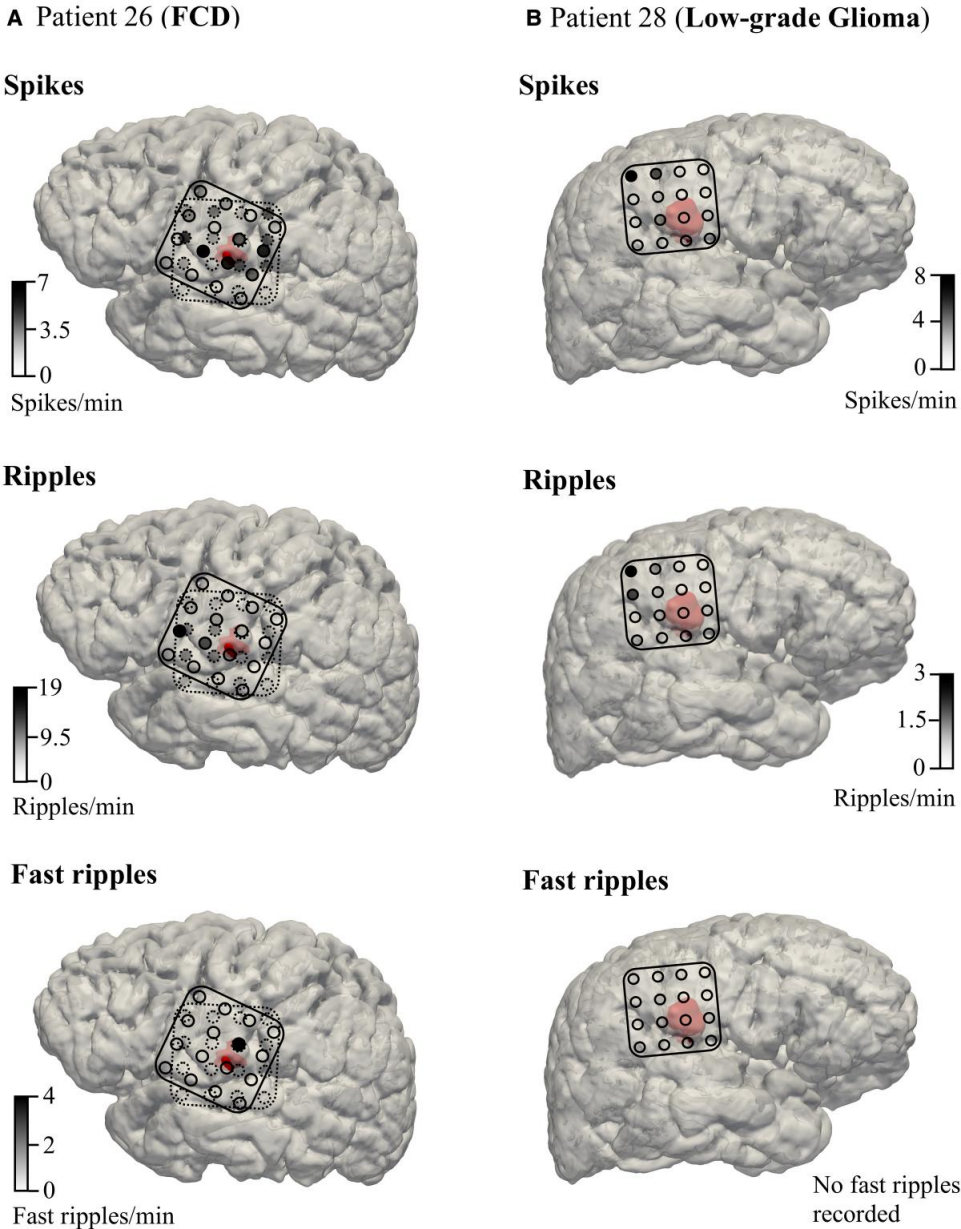
**Illustrative FCD and low-grade glioma cases.** (**A**) Patient 26 (FCD, left) and (**B**) Patient 28 (low-grade glioma, right) are portrayed to illustrate the typical ioECoG findings for these pathology types in our study. Two presurgical ioECoG recordings were taken for Patient 26 (solid and dashed lines) and one was taken for Patient 28 (solid line). Electrodes with high spike, ripple and fast ripple rates are located close to the MRI lesion (red segmentation) for the FCD patient. Conversely, electrodes with high spike and ripple rates are far away from the MRI lesion for the low-grade glioma patient. No fast ripples were recorded for Patient 28.

We illustrate this finding in Fig. 6, where we demonstrate our results in an FCD and a low-grade glioma case. Our analyses suggest that high spike, ripple and fast ripple rates are closely linked to FCD borders, as is the case in the FCD patient in Fig. 6A. Since FCDs most likely generate epileptic activity intrinsically,^18,57–59^ the presence of high spike/HFO rates in the absence of a clear tissue border may indicate lesional tissue not visible on regular 3T MRI. This advocates for the resection of the tissue underneath these electrodes. Without ioECoG tailoring, this tissue might be missed during surgical resection. This notion is supported by earlier studies that have shown ioECoG-tailored lesionectomy to be linked to a better surgical outcome than lesionectomy alone in FCDs.^56,66^

While not a direct subject of our analyses, we believe this argues for the potential usefulness of stereoelectroencephalography (SEEG) in FCD patients. SEEG may delineate intrinsically epileptogenic FCD tissue at the bottom of the sulcus, which ioECoG may not adequately capture.^67^ However, SEEG provides highly localized data, which may not be adequate to delineate the entire edge of the lesion. In our opinion, it is probably most useful as an addition to ioECoG.

In low-grade gliomas, on the other hand, epileptiform activity was most often found far outside the radiographic lesion, as is the case in the exemplary patient in Fig. 6B. Whether the removal of such distant foci is also related to good surgical outcomes is debated. Some studies have found that ioECoG-tailored resections yield better seizure control for tumour-related focal epilepsy than lesionectomy alone,^68,69^ while others have not.^70,71^ That tumourous and non-tumourous lesions seem to differ in that regard illustrates that the (presumed) pathology type should be considered when ioECoG results are used to tailor the resection.

That high spike/ripple rates appear to be a good indicator of proximity to the lesion edge in FCDs is also potentially useful to determine the site of corticotomy at the beginning of a resection, especially when the precise location of the radiographic lesion is not immediately apparent due to brain shift or other uncertainties. However, our data suggest care should be taken with this approach for tumourous lesions, especially low-grade gliomas. High spike/HFO rates may not correlate with proximity to the lesion in these patients.

Ultimately, our data suggest that ioECoG biomarkers can provide different information to the neurosurgeon depending on the underlying lesion type. Future research is needed to confirm these findings and focus on implementing them into a clinical guideline on how to interpret ioECoG data by pathology type. Frauscher *et al*.^72^ recently proposed an atlas with normative values for physiological HFO rates according to different anatomical locations. We suggest that the influence of pathology types on ioECoG results may be a useful addition to such an atlas.

### Strengths and potential limitations of this study

A strength of our study is its novelty. To our knowledge, this is the first study investigating correlations between neocortical spike/HFO rates and distance as a measure of their spatial relationship to MRI lesions. We hope future studies can benefit from our methods to overcome similar technical hurdles in fusing MRI and ioECoG data.

A limitation of our study is its retrospective design and the relatively small number of included patients. These small numbers are in part due to our strict inclusion criteria to minimize confounding factors. We excluded all patients with non-optimal surgical outcome, incomplete resections and repeated surgeries because these factors might indicate that the EZ extended further than the area on which ioECoG was performed. It would be interesting for future studies to compare the spatial relationship between MRI and ioECoG data between patients who achieved postoperative seizure freedom and those who did not. Potentially, patients with an unfavourable outcome might display a different relationship than the one we found in this study, or none at all. A comparative study design might further improve our understanding of the reason why some patients do not achieve seizure freedom. It would also be of interest to compare the MRI/ioECoG relationship between resected and non-resected electrodes in such a future comparative study.

We furthermore excluded patients with mesiotemporal lesions because mesiotemporal structures have been shown to display higher HFO rates than superficial lesions.^19^ Inclusion of mesiotemporal lesions might therefore have confounded our analyses.

A limitation of our analyses is the possible inclusion of physiological along with pathological ripples.^30^ We marked spikes, ripples and fast ripples separately to ensure that markings were not co-dependent on each other. This may have increased the difficulty in distinguishing between physiological and pathological ripples during marking.^28^

An important limitation of our study is that we identified few fast ripples and none for three pathology types. Because fast ripples usually occur at a relatively slow rate,^19,34^ the analysis of a 1 min epoch per ioECoG recording may not have been sufficient for an accurate representation of the rate of fast ripples. We therefore additionally analysed the correlation between distance and fast ripple *occurrence* (as a dichotomous variable) rather than fast ripple *rates*. While we found no significant correlation, fast ripples showed a marginally significant tendency to occur closer to the lesion edge in FCDs. This supports the notion that FCDs generate epileptogenicity intrinsically.^18,57–59^

While ripples also occur in the physiological brain and not only in epileptogenic tissue, fast ripples are considered mostly pathological and are therefore specific markers for epileptogenicity.^19,34^ The use of high-density ioECoG grids or additional depth electrodes in future studies may increase the detection of fast ripples.^44,73^

The manual placement of visible electrodes on the cortical reconstruction using prominent sulci and vessels as guidelines and the determination of the spatial midpoint between two electrode markers as the bipolar electrode position naturally led to some spatial error. We believe that this error is sufficiently small for our analyses. Although estimating this error is difficult without access to the actual position of the electrode, we estimate it to be no more than 2–3 mm, probably less for most electrodes. Further errors in measurement such as during the marking of electrographic events, and the limitation to 1 min ioECoG epochs may have also influenced our analyses.

We did not correct for multiple comparisons for our linear regression results. However, the application of a Bonferroni correction would not have significantly altered our results.

In most patients, the ioECoG was recorded from two sides around the lesion. In some, only one or more than two recordings were performed, spread out over the surgical situs. This depended on the size and location of the lesion and on practical issues. We included all recordings for all patients without correcting for multiple recordings. A potential limitation of this methodology is that it may have introduced bias towards finding more electrographic events in patients with multiple recordings than in patients with few recordings. However, we assumed all recordings to be necessary to cover the entire EZ. Not including all recordings might thus have led to a selection bias and/or exclusion of a part of the EZ for these patients. Introducing artificial weighting factors might have reduced the accuracy of our data set and/or favoured patients with fewer recordings. Hence, we believed it most prudent to work with our data unaltered.

It should also be noted that propofol, the hypnotic agent used during all surgeries, has been shown to reduce the HFO rates, while spike rates remain largely unaffected.^40^ To counteract this, the administration of propofol was discontinued until a continuous ioECoG background pattern was reached (5–10 min) before ioECoG recordings were taken. We believe this discontinuation to be sufficient for an accurate measurement of HFO rates; still, a remaining suppressive effect of propofol cannot be entirely ruled out.

Our retrospective process of marking individual spikes and HFOs differs from the method used in the HFO trial, where spikes and HFOs were marked prospectively, according to general occurrence rather than exact rates, and using different software than used for this study.

We focused solely on spike and HFO rates as indicators of epileptogenicity in this study, with no further classification. Several studies indicate that tailoring the resection to tissue generating *leading* instead of *propagated* spikes might be favourable to removing all tissue where spikes are recorded.^5,71,74,75^ HFOs are also thought to propagate from their area of generation,^13^ and the resection of leading ripples may similarly be associated with better postsurgical seizure control than the resection of propagated ripples.^32^ It would be interesting to investigate the spatial relationship specifically between leading spikes/ripples and the MRI lesion in a future study.

We would like to encourage future studies on spikes and HFOs to perform subgroup analyses stratified by pathology type. This may advance our understanding of the influence of pathology type, particularly tumourous versus non-tumourous lesions, on ioECoG results.

## Supplementary Material

fcac302_Supplementary_Data

## Data Availability

The custom Java scripts required for the placement and extrapolation of electrode markers on the cortical surface in Java Navigation (release 2021) can be accessed via the public repository GitHub (https://github.com/UMCU-EpiLAB/umcuEpi_MRI_and_ioECoG). The entire software and the data that support our findings can be made available upon reasonable request to the corresponding author.

## Abbreviations

CI =: confidence interval
COM =: centre of mass
DNET =: dysembryoplastic neuroepithelial tumour
EZ =: epileptogenic zone
FCD =: focal cortical dysplasia
FIR filter =: finite impulse response filter
FLAIR =: fluid attenuated inversion recovery
HFO =: high-frequency oscillation
ioECoG =: intraoperative electrocorticography
OR =: odds ratio
PXA =: pleomorphic xanthoastrocytoma
SD =: standard deviation
