# Supplementary material for: The spatial relationship between the MRI lesion and intraoperative electrocorticography in focal epilepsy surgery
Source: Brain Commun. 2022 Nov 21;4(6):fcac302. doi: 10.1093/braincomms/fcac302 (PMC9732864; doi:10.1093/braincomms/fcac302)
Supplement: fcac302_Supplementary_Data [file fcac302_supplementary_data.zip › Supplementary_File_1.docx]

**Supplementary Material 1 Presurgical MRIs for illustrative cases for all pathology types with and without a segmentation of the MRI lesion**

Screenshots were taken from the program *ITK-SNAP*, which was used to create the segmentations of all lesions.

**FCD**

**
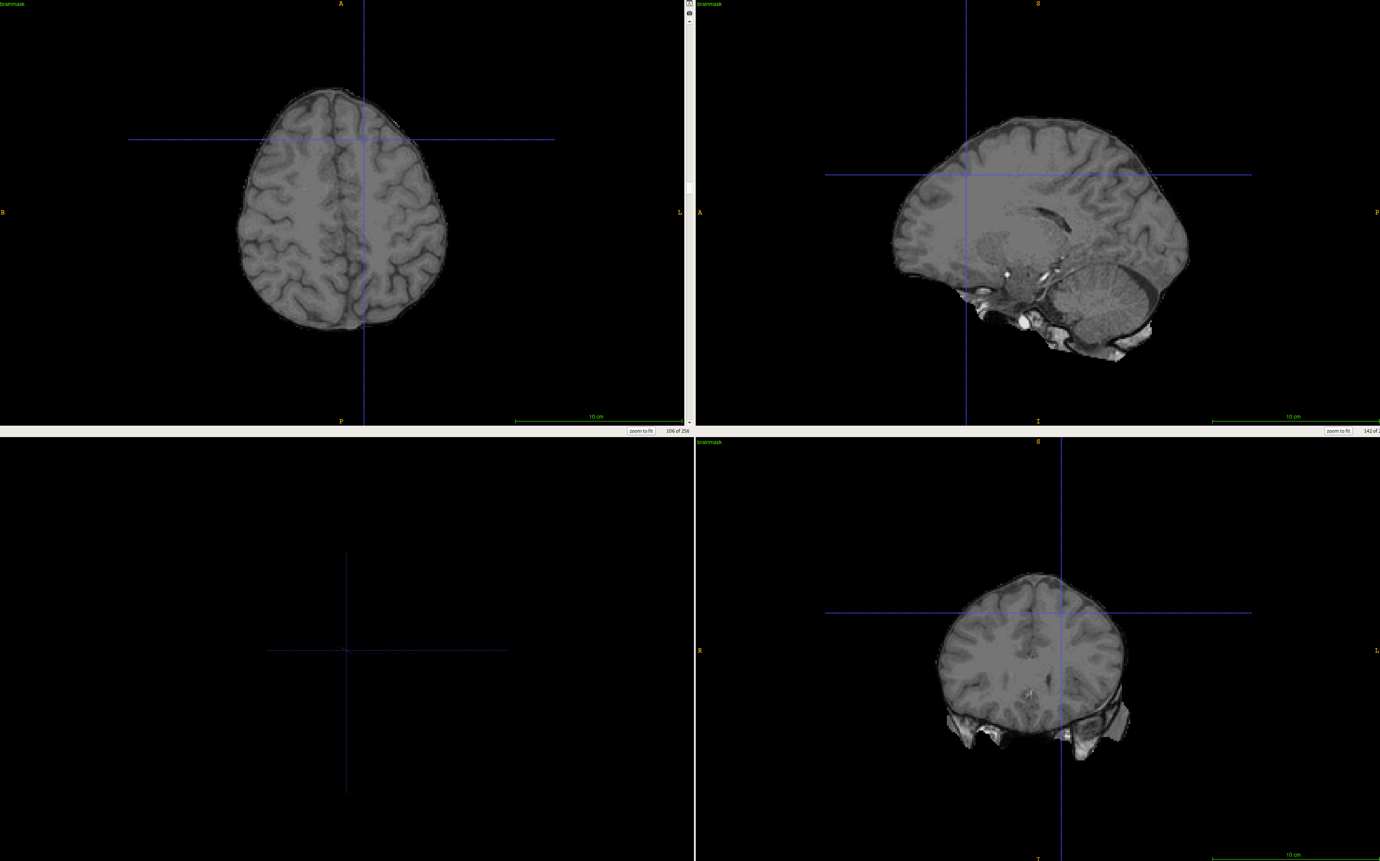

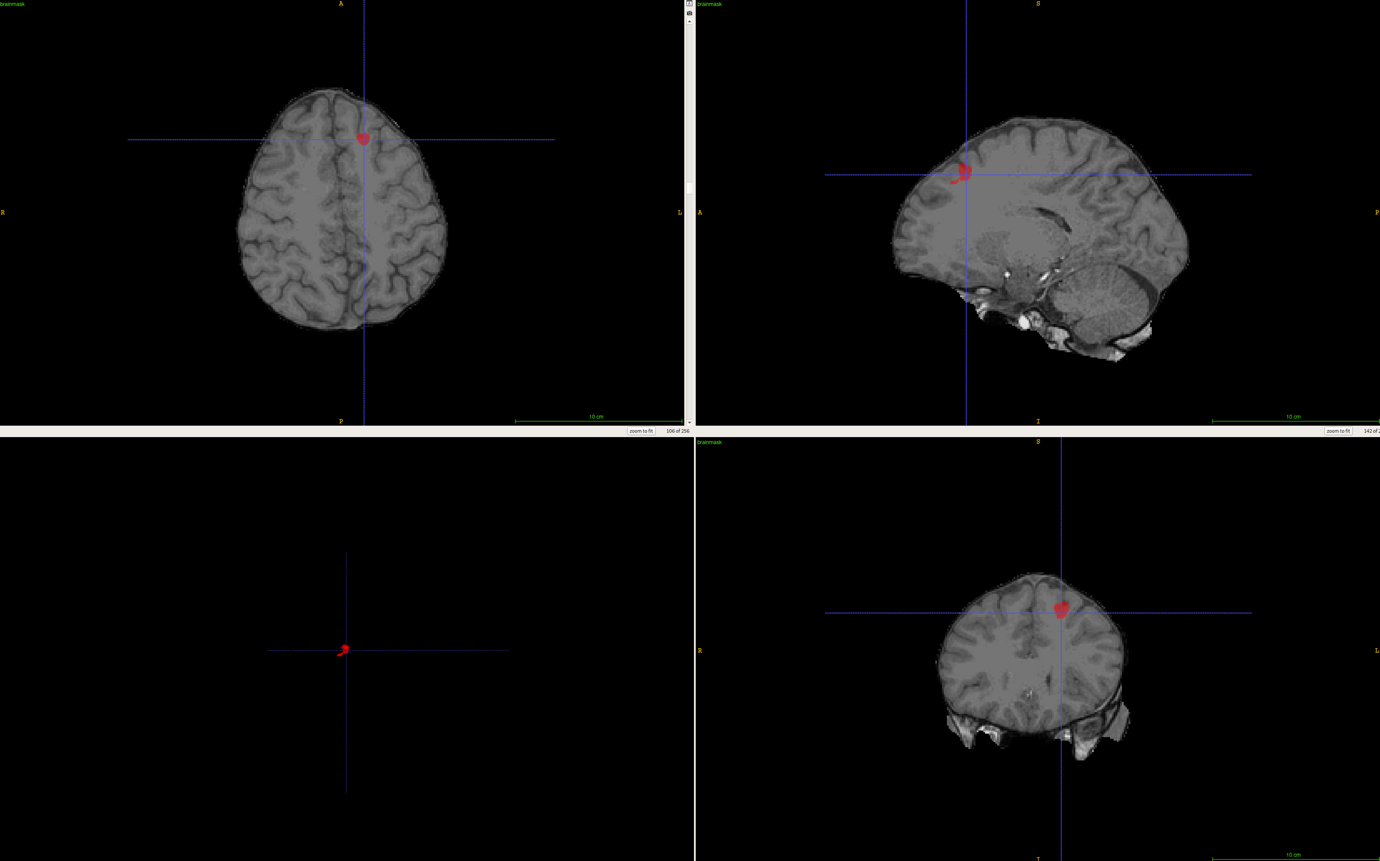
**

**Ganglioglioma**

**
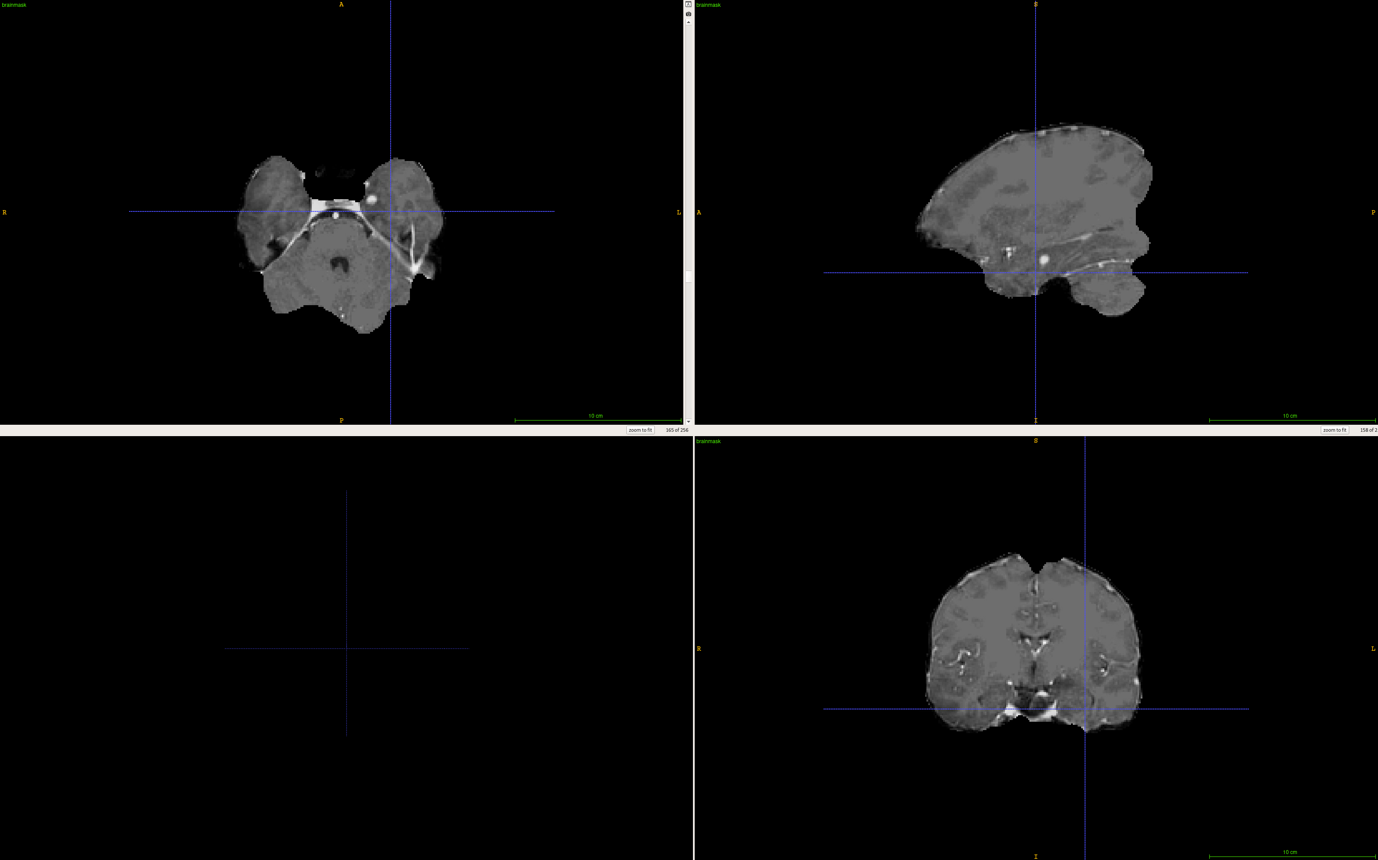

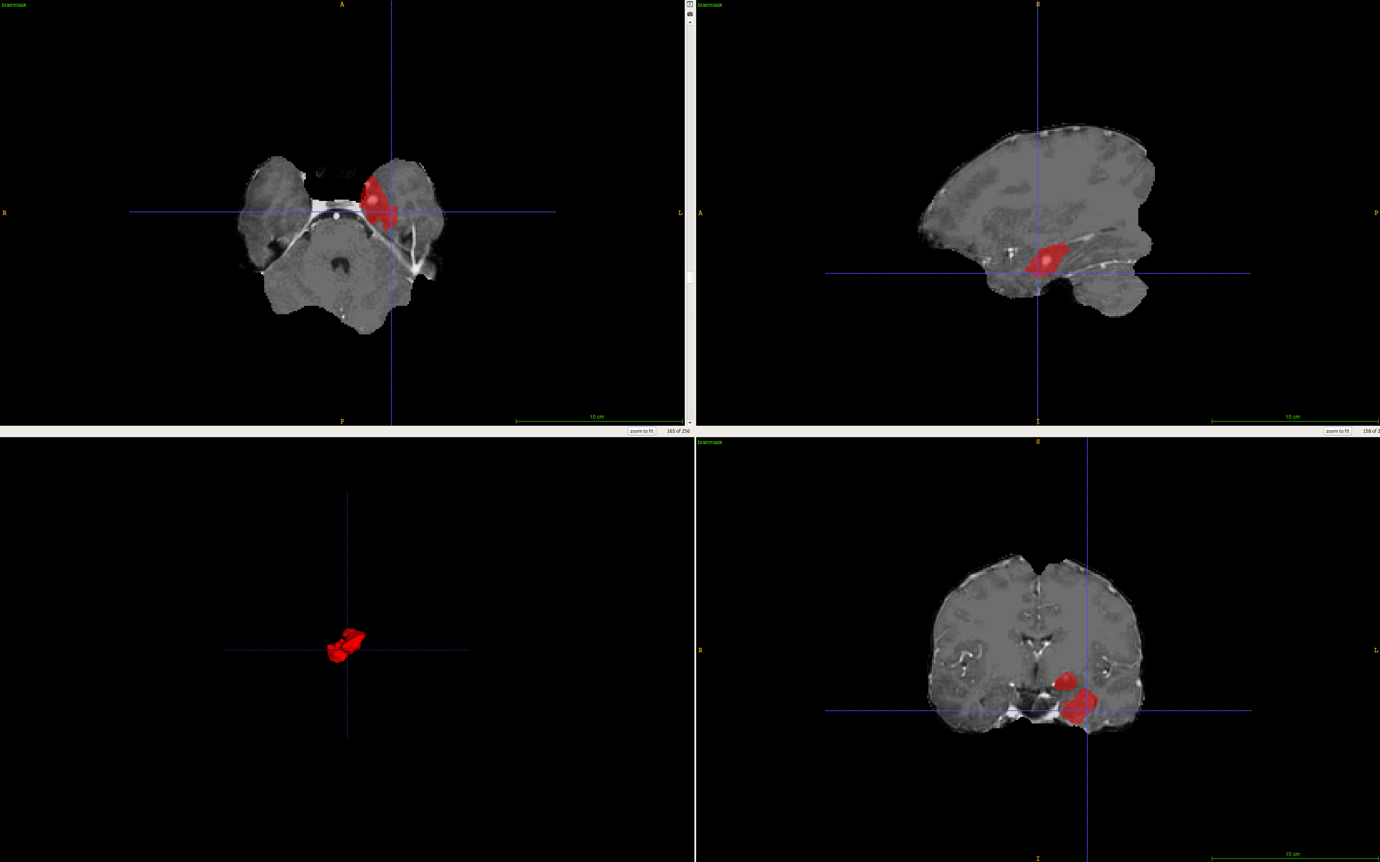
**

**DNET**

**
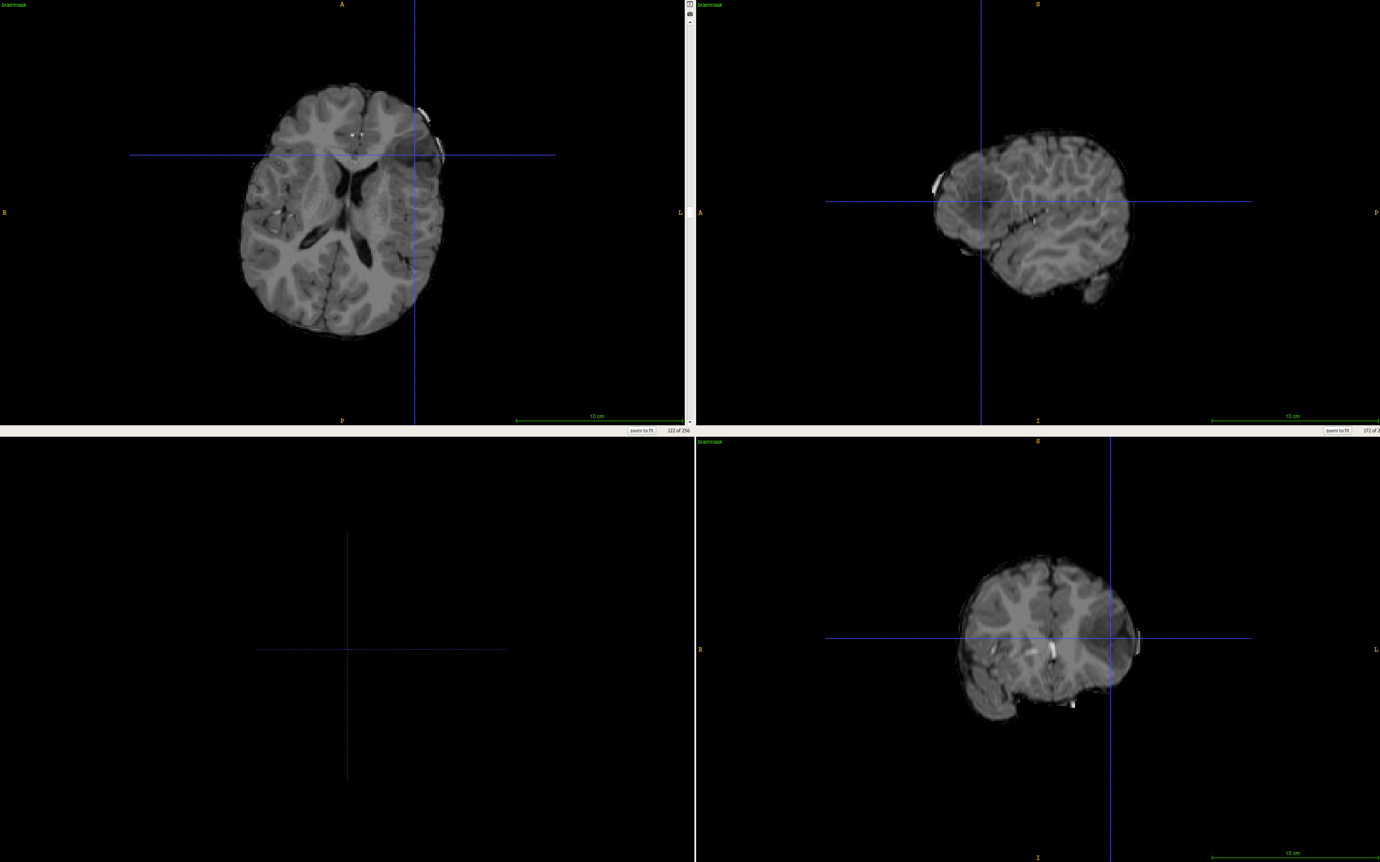

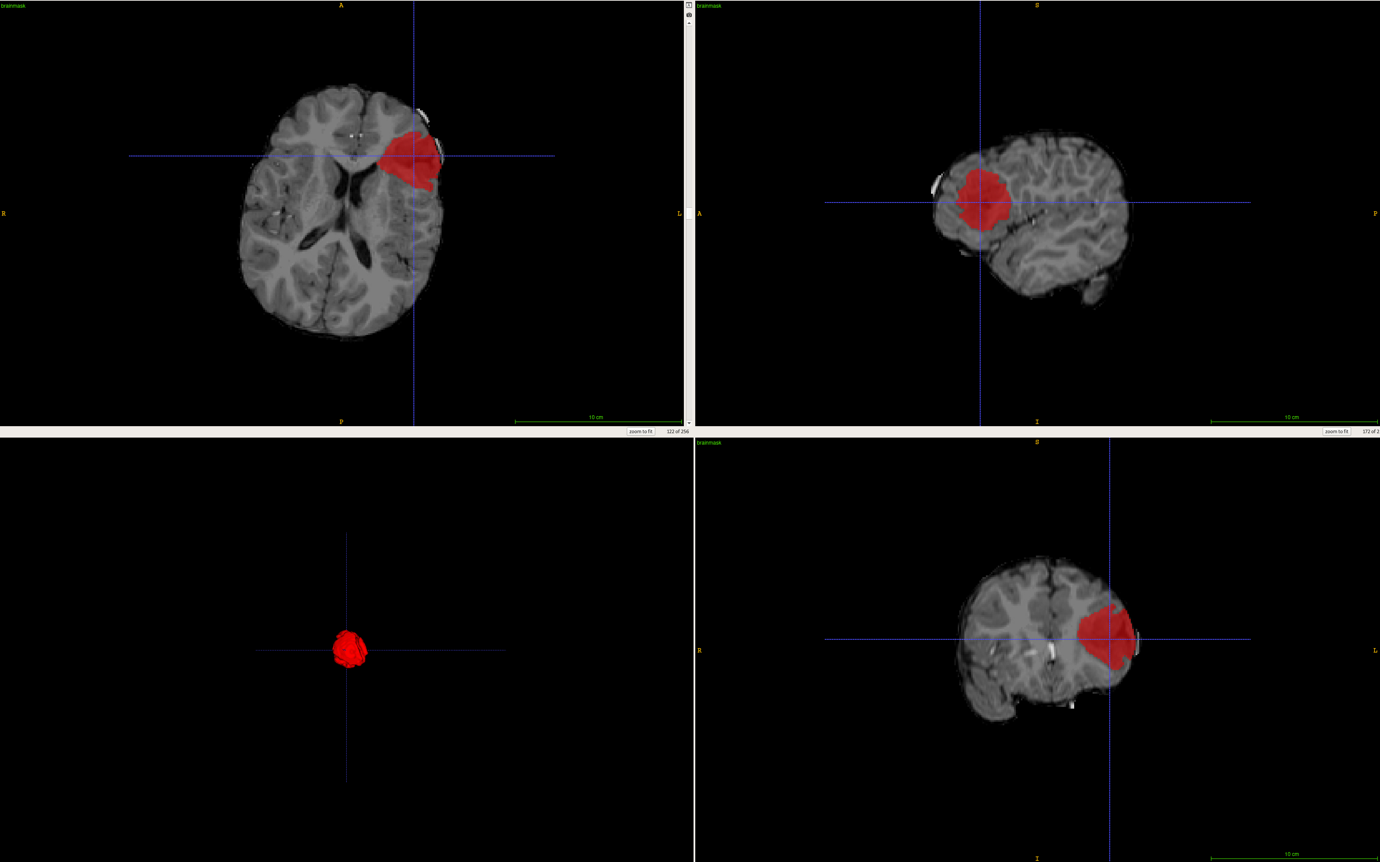
**

**Low-grade Glioma**

**
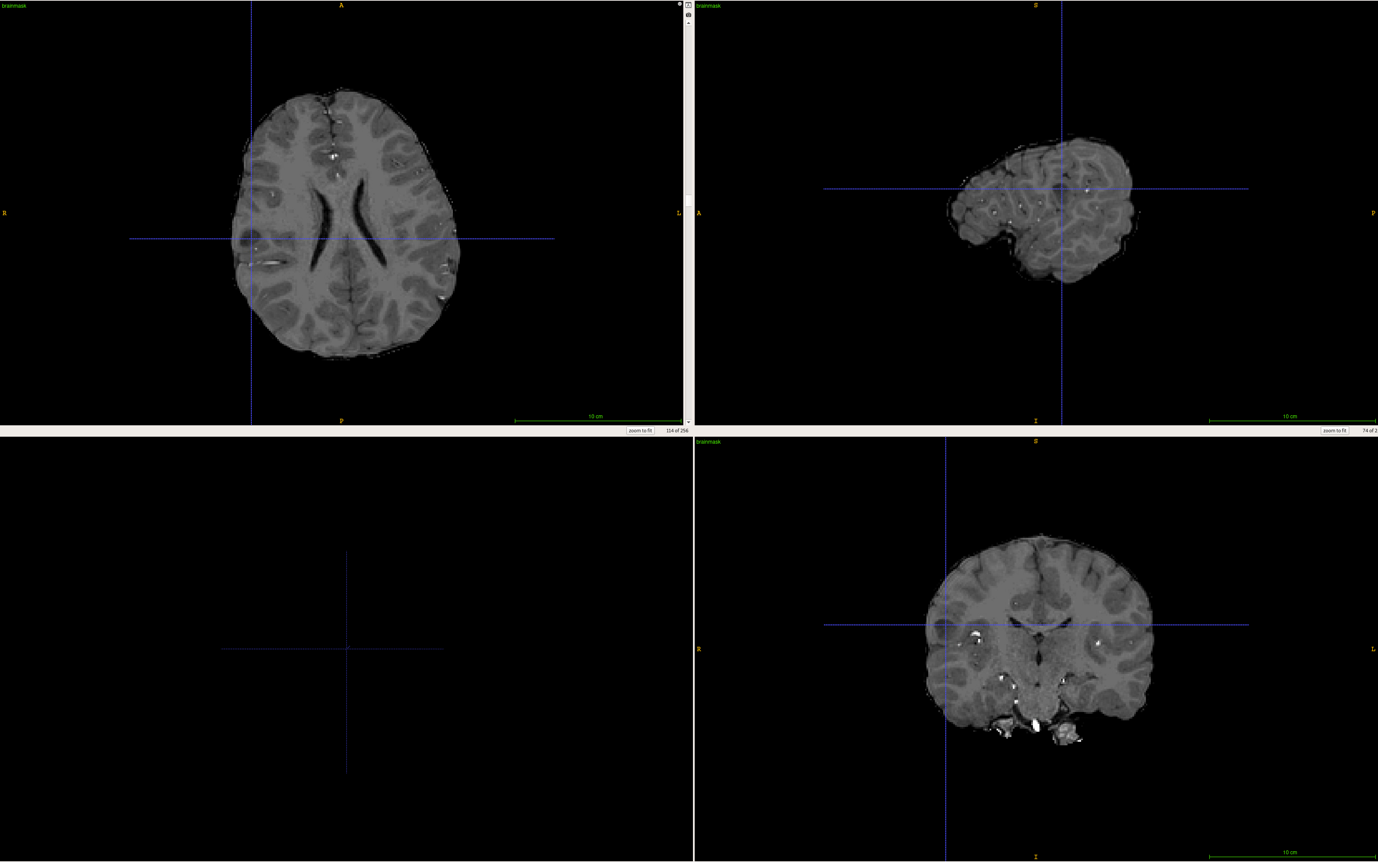

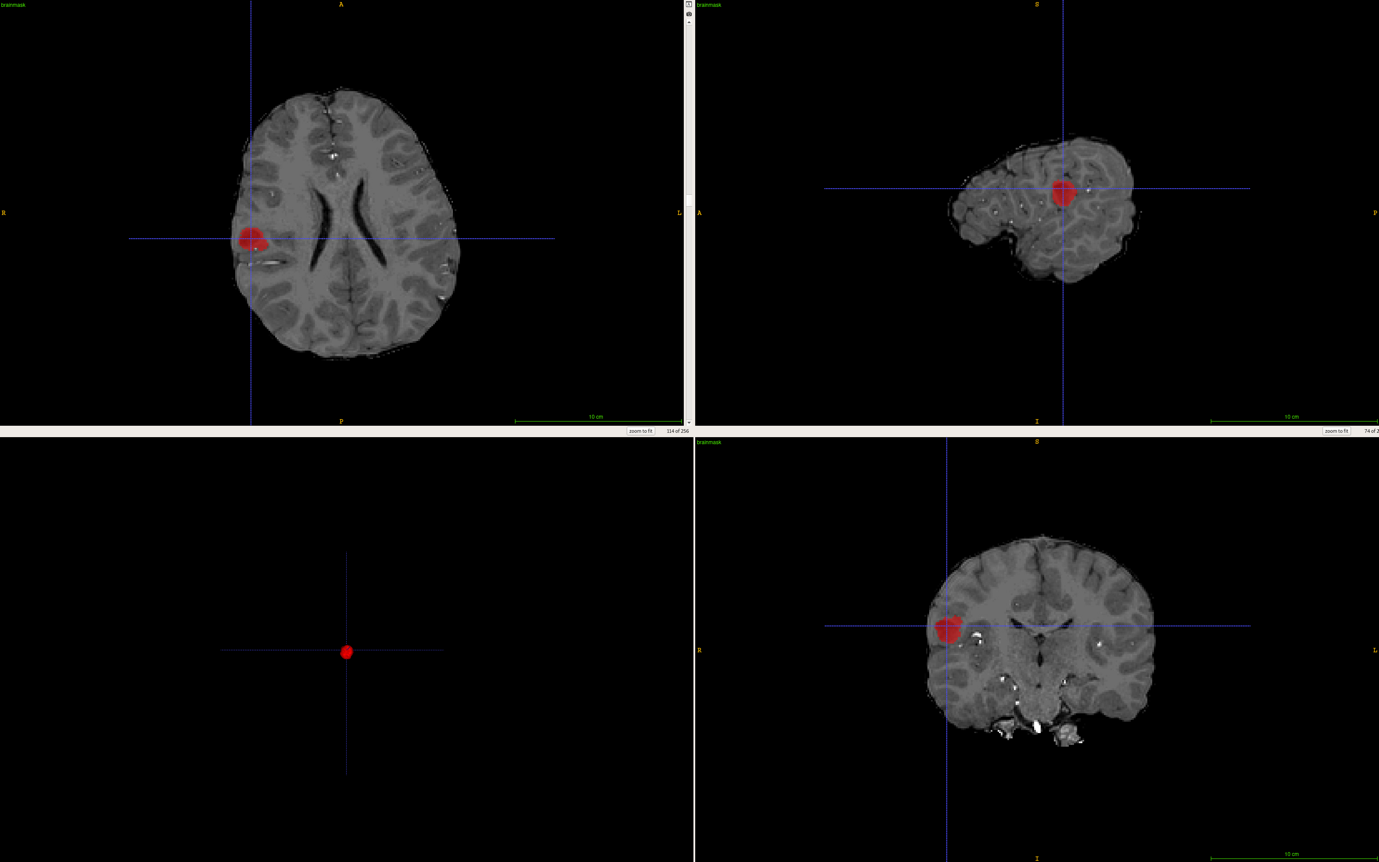
**

**PXA**

**
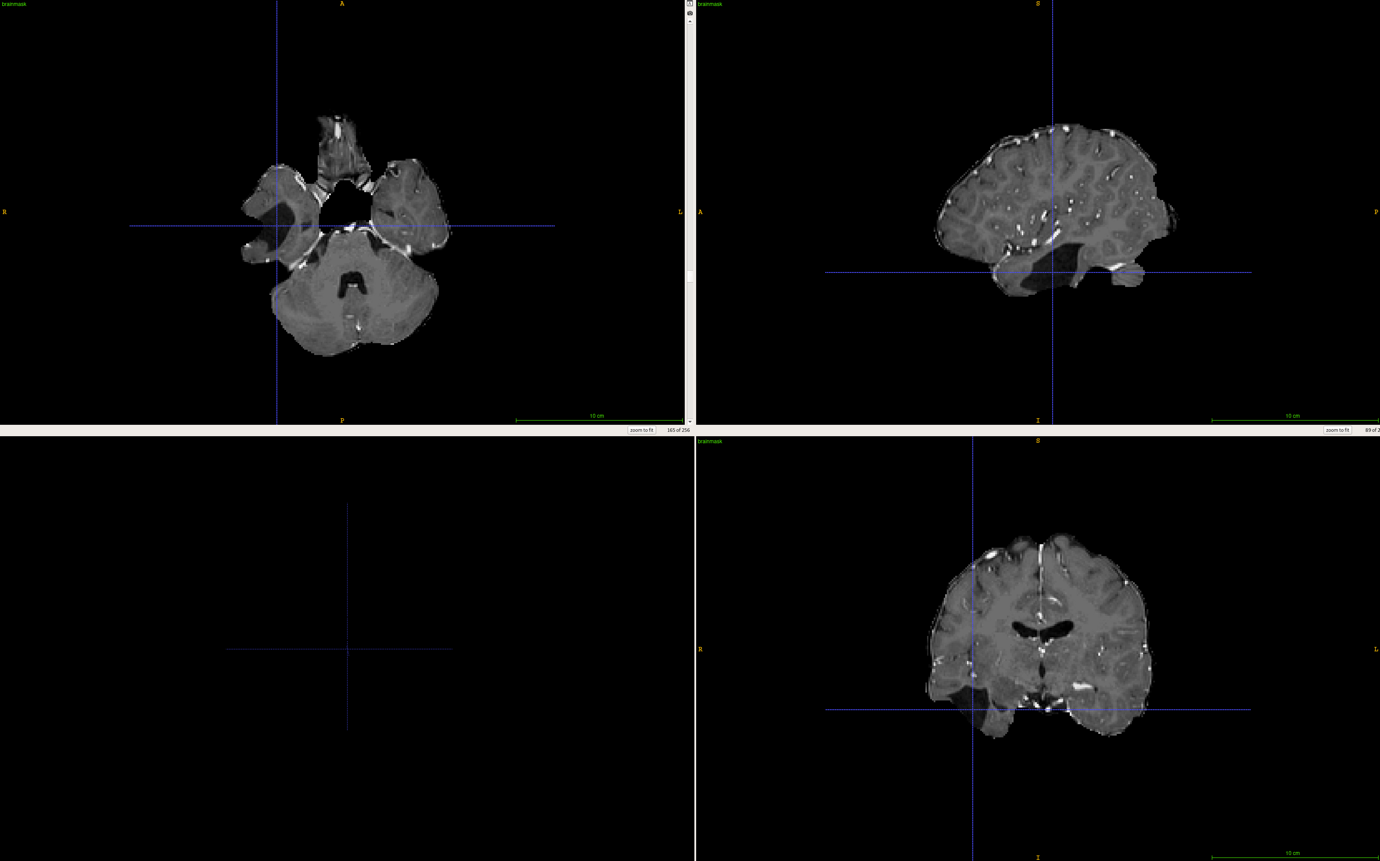

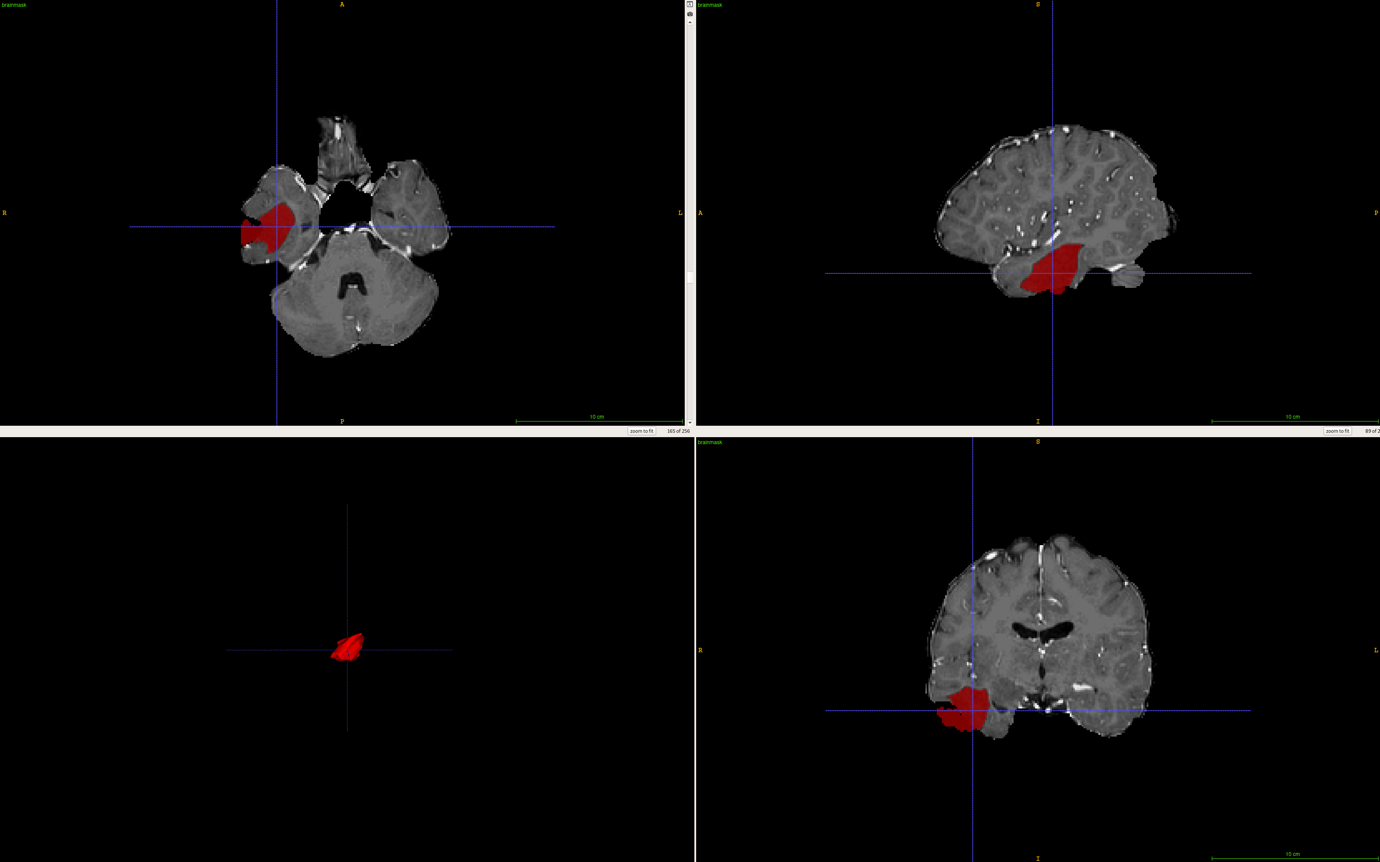
**

**Cavernoma**

**
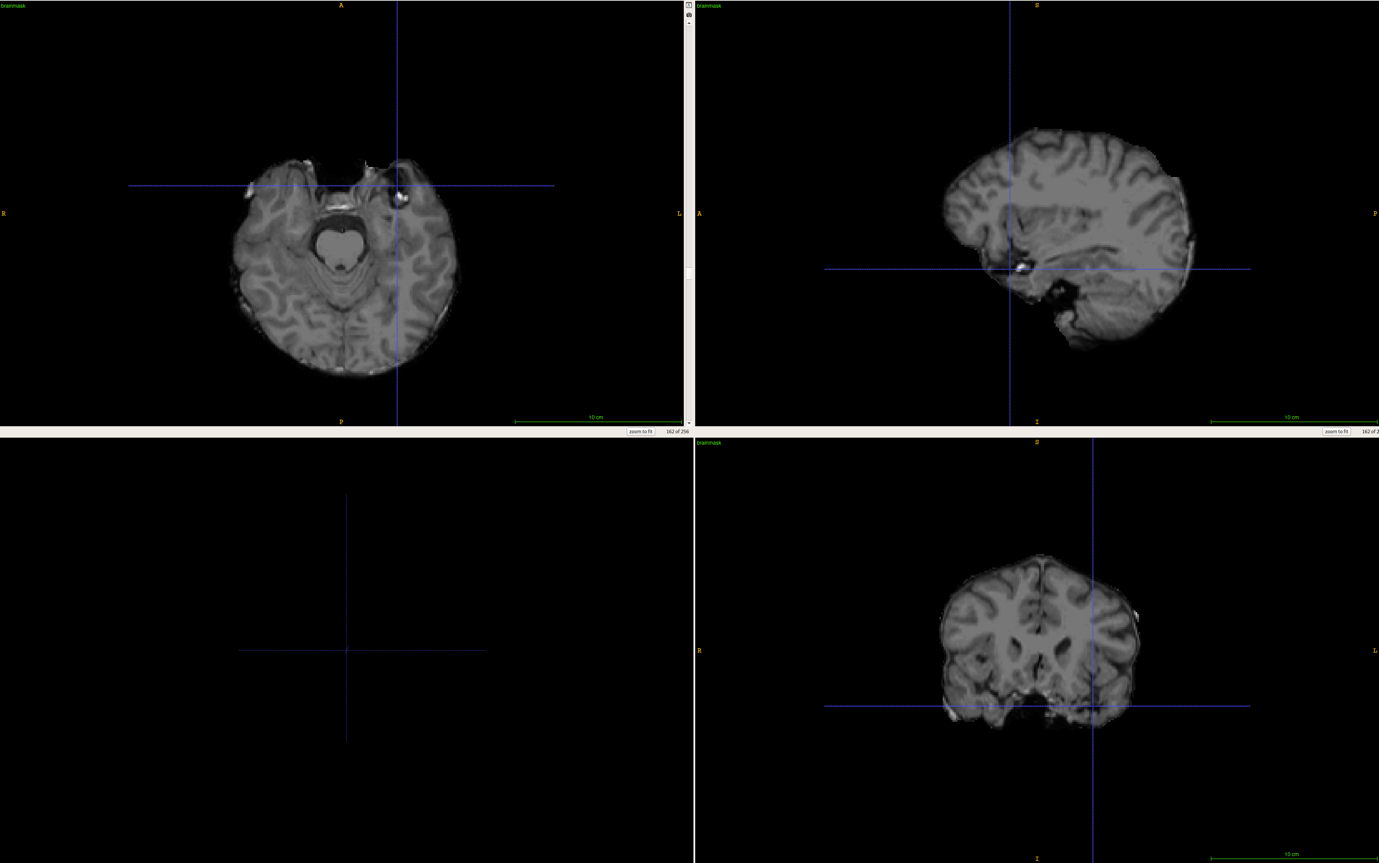

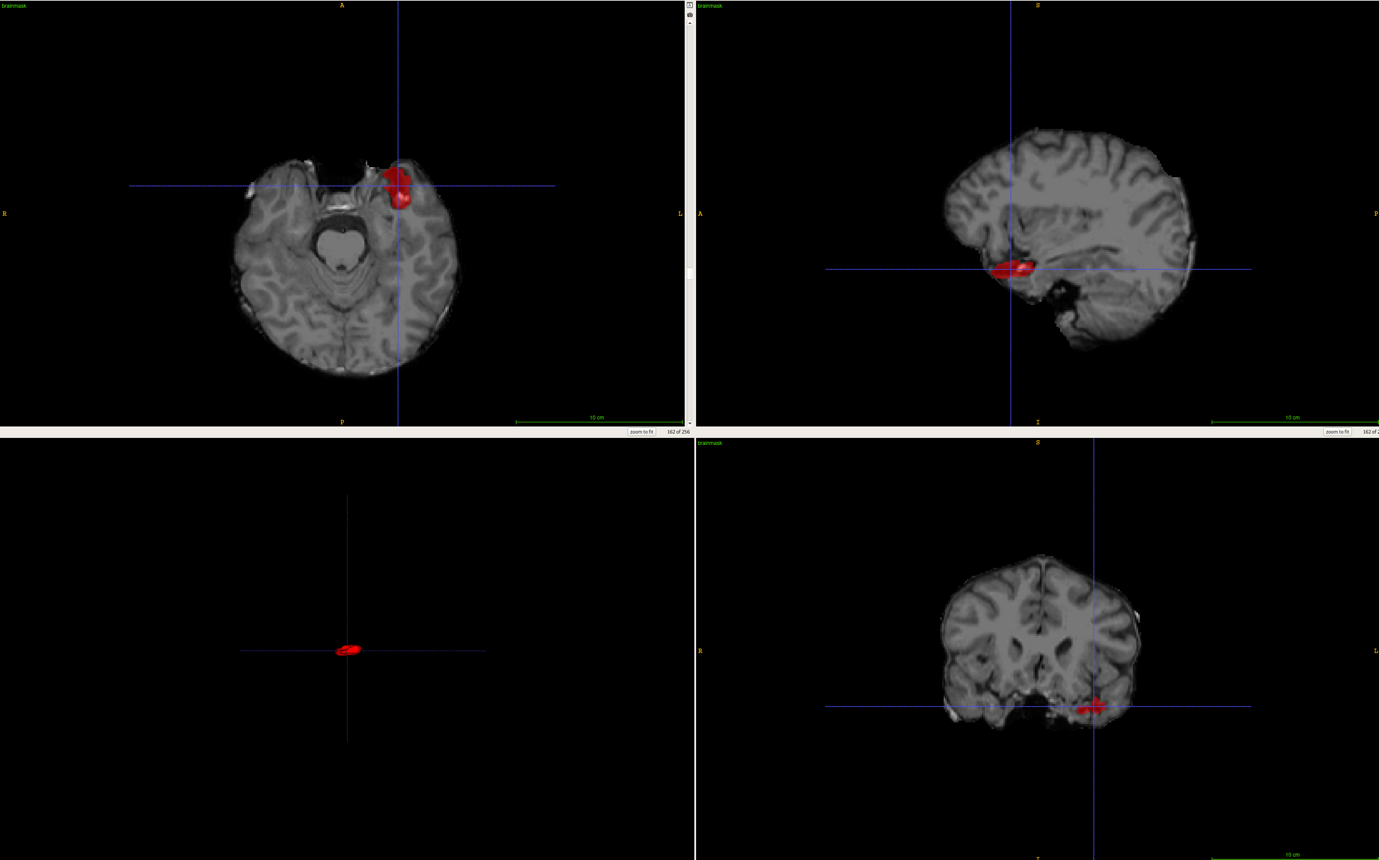
**
