# Supplementary material for: The spatial relationship between the MRI lesion and intraoperative electrocorticography in focal epilepsy surgery
Source: Brain Commun. 2022 Nov 21;4(6):fcac302. doi: 10.1093/braincomms/fcac302 (PMC9732864; doi:10.1093/braincomms/fcac302)
Supplement: fcac302_Supplementary_Data [file fcac302_supplementary_data.zip › Supplementary_Tables.docx]

|  |  | (1) Distance to the edge of the lesion | | | | | (2) Distance to the centre of the lesion | | | | |
| --- | --- | --- | --- | --- | --- | --- | --- | --- | --- | --- | --- |
|  | Subgroup | *B* | *HCSE* | *p* | *p*(bs) | *η^2^* | *B* | *HCSE* | *p* | *p*(bs) | *η^2^* |
| Spike rates | All patients (*n*=33) | **-0.10** | **0.04** | **0.03** | **0.03** | **0.004** | -0.10 | 0.05 | 0.06 | 0.05 | 0.003 |
|  | Cavern. (*n*=2) | **-1.37** | **0.32** | **<0.0001** | **0.005** | **0.22** | **-1.49** | **0.32** | **<0.0001** | **0.003** | **0.26** |
|  | DNET (*n*=5) | -0.35 | 0.29 | 0.23 | 0.21 | 0.006 | -0.27 | 0.34 | 0.43 | 0.44 | 0.003 |
|  | FCD (*n*=12) | **-0.25** | **0.05** | **<0.0001** | **0.001** | **0.05** | **-0.21** | **0.05** | **<0.0001** | **0.001** | **0.03** |
|  | GG (*n*=9) | 0.09 | 0.10 | 0.38 | 0.33 | 0.002 | 0.17 | 0.12 | 0.16 | 0.12 | 0.005 |
|  | LGG (*n*=3) | **0.65** | **0.10** | **<0.0001** | **0.001** | **0.37** | **0.39** | **0.13** | **0.005** | **0.006** | **0.10** |
|  | PXA (*n*=2) | **-0.18** | **0.07** | **0.01** | **0.02** | **0.09** | -0.07 | 0.09 | 0.42 | 0.38 | 0.01 |
| Ripple rates | All patients (*n*=33) | **-0.14** | **0.07** | **0.04** | **0.04** | **0.003** | **-0.22** | **0.07** | **0.003** | **0.004** | **0.01** |
|  | Cavern. (*n*=2) | -1.04 | 2.65 | 0.70 | 0.60 | 0.002 | -1.73 | 2.76 | 0.53 | 0.41 | 0.01 |
|  | DNET (*n*=5) | 0.32 | 0.55 | 0.56 | 0.37 | 0.001 | 0.38 | 0.80 | 0.64 | 0.50 | 0.001 |
|  | FCD (*n*=12) | **-0.35** | **0.07** | **<0.0001** | **0.001** | **0.05** | **-0.33** | **0.07** | **<0.0001** | **0.001** | **0.04** |
|  | GG (*n*=9) | 0.04 | 0.16 | 0.81 | 0.80 | 0.001 | 0.04 | 0.14 | 0.78 | 0.78 | 0.001 |
|  | LGG (*n*=3) | **2.67** | **0.58** | **<0.0001** | **0.002** | **0.22** | **1.46** | **0.53** | **0.008** | **0.02** | **0.09** |
|  | PXA (*n*=2) | 0.08 | 0.55 | 0.88 | 0.82 | 0.001 | 0.41 | 1.02 | 0.69 | 0.47 | 0.002 |
| Fast ripple rates | All patients (*n*=33) | -0.57 | 0.58 | 0.33 | 0.004 | 0.001 | -0.92 | 1.18 | 0.43 | 0.003 | 0.001 |
|  | Cavern. (*n*=2) | N/A^a^ | - | - | - | - | N/A^a^ | - | - | - | - |
|  | DNET (*n*=5) | N/A^a^ | - | - | - | - | N/A^a^ | - | - | - | - |
|  | FCD (*n*=12) | -0.58 | 0.65 | 0.37 | 0.01 | 0.001 | -0.79 | 1.06 | 0.46 | 0.007 | 0.001 |
|  | GG (*n*=9) | **-2.22** | **0.68** | **0.001** | **0.003** | **0.03** | **-2.19** | **0.64** | **0.001** | **0.005** | **0.03** |
|  | LGG (*n*=3) | N/A^a^ | - | - | - | - | N/A^a^ | - | - | - | - |
|  | PXA (*n*=2) | -2.10 | 6.53 | 0.75 | N/A^b^ | 0.002 | 2.13 | 4.53 | 0.64 | N/A^b^ | 0.003 |

**Supplementary Table 1 Linear regressions for spike, ripple, and fast ripple rates as predictors for distance**

|  |  | (3) Distance along the cortical surface | | | | |
| --- | --- | --- | --- | --- | --- | --- |
|  | Subgroup | *B* | *HCSE* | *p* | *p*(bs) | *η^2^* |
| Spike rates | All patients (*n*=33) | -0.10 | 0.10 | 0.34 | 0.33 | 0.001 |
|  | Cavernoma (*n*=2) | 2.00 | 1.79 | 0.27 | 0.04 | 0.02 |
|  | DNET (*n*=5) | **-1.18** | **0.51** | **0.02** | **0.02** | **0.02** |
|  | FCD (*n*=12) | 0.12 | 0.16 | 0.47 | 0.48 | 0.001 |
|  | Ganglioglioma (*n*=9) | -0.15 | 0.13 | 0.22 | 0.19 | 0.005 |
|  | LG Glioma (*n*=3) | -0.08 | 0.54 | 0.88 | 0.84 | 0.000 |
|  | PXA (*n*=2) | **-1.20** | **0.38** | **0.003** | **0.004** | **0.23** |
| Ripple rates | All patients (*n*=33) | -0.05 | 0.13 | 0.71 | 0.73 | 0.000 |
|  | Cavernoma (*n*=2) | -1.11 | 3.32 | 0.74 | 0.65 | 0.002 |
|  | DNET (*n*=5) | -1.47 | 1.09 | 0.18 | 0.07 | 0.007 |
|  | FCD (*n*=12) | 0.37 | 0.19 | 0.05 | 0.04 | 0.01 |
|  | Ganglioglioma (*n*=9) | -0.11 | 0.21 | 0.61 | 0.59 | 0.001 |
|  | LG Glioma (*n*=3) | -3.20 | 1.62 | 0.05 | 0.05 | 0.05 |
|  | PXA (*n*=2) | -0.75 | 2.35 | 0.75 | 0.67 | 0.003 |
| Fast ripple rates | All patients (*n*=33) | -0.45 | 1.07 | 0.68 | 0.63 | <0.001 |
|  | Cavernoma (*n*=2) | N/A^a^ | - | - | - | - |
|  | DNET (*n*=5) | N/A^a^ | - | - | - | - |
|  | FCD (*n*=12) | 0.32 | 1.27 | 0.80 | 0.74 | <0.001 |
|  | Ganglioglioma (*n*=9) | 1.34 | 4.61 | 0.77 | 0.64 | <0.001 |
|  | LG Glioma (*n*=3) | N/A^a^ | - | - | - | - |
|  | PXA (*n*=2) | **-35.40** | **7.56** | **<0.001** | **N/A^b^** | **0.39** |

Univariate linear regressions for spike, ripple, and fast ripple rates as predictors for the distances (1), (2), and (3), outlined in the *materials and methods*. Linear regressions were calculated for all patients combined, and stratified by pathology. Statistically significant models are highlighted in bold. Abbreviations: dysembryoplastic neuroepithelial tumor (DNET), focal cortical dysplasia (FCD), ganglioglioma (GG), Low-grade glioma (LGG), pleomorphic xanthoastrocytoma (PXA), cavernoma (cavern.), coefficient *B*, heteroscedasticity consistent standard error (*HCSE*), *p*-value, *p-*value bootstrap (*bs*), and effect size *η^2^.*

^a^No fast ripples were recorded for this pathology. ^b^Not enough fast ripples were recorded for PXAs to perform bootstrapping.

| Pathology Type | *At Lesion Volume (cm^3^)* | *Effect* | *HCSE* | *t* | *p* | *LLCI* | *ULCI* |
| --- | --- | --- | --- | --- | --- | --- | --- |
| Cavernoma (*n*=2) | **0.5** | **-1.37** | **0.32** | **-4.26** | **<0.0001** | **-2.00** | **-0.74** |
| Cavernoma (*n*=2) | **10.4** | **-1.34** | **0.33** | **-4.10** | **<0.0001** | **-1.98** | **-0.70** |
| Cavernoma (*n*=2) | **22.5** | **-1.30** | **0.35** | **-3.7** | **0.0002** | **-1.99** | **-0.61** |
| DNET (*n*=5) | 0.5 | -0.39 | 0.31 | -1.25 | 0.21 | -1.00 | 0.22 |
| DNET (*n*=5) | 10.4 | -0.36 | 0.29 | -1.22 | 0.22 | -0.93 | 0.22 |
| DNET (*n*=5) | 22.5 | -0.32 | 0.29 | -1.09 | 0.28 | -0.89 | 0.26 |
| FCD (*n*=12) | **0.5** | **-0.27** | **0.05** | **-5.06** | **<0.0001** | **-0.37** | **-0.16** |
| FCD (*n*=12) | **10.4** | **-0.23** | **0.06** | **-3.79** | **0.0002** | **-0.36** | **-0.11** |
| FCD (*n*=12) | 22.5 | -0.19 | 0.13 | -1.45 | 0.15 | -0.46 | 0.07 |
| GG (*n*=9) | 0.5 | 0.05 | 0.14 | 0.33 | 0.74 | -0.23 | 0.32 |
| GG (*n*=9) | 10.4 | 0.08 | 0.11 | 0.74 | 0.46 | -0.13 | 0.28 |
| GG (*n*=9) | 22.5 | 0.12 | 0.11 | 1.02 | 0.31 | -0.11 | 0.34 |
| LG Glioma (*n*=3) | **0.5** | **0.53** | **0.10** | **5.20** | **<0.0001** | **0.33** | **0.73** |
| LG Glioma (*n*=3) | **10.4** | **0.56** | **0.10** | **5.59** | **<0.0001** | **0.37** | **0.76** |
| LG Glioma (*n*=3) | **22.5** | **0.60** | **0.15** | **4.00** | **0.0001** | **0.31** | **0.90** |
| PXA (*n*=2) | 0.5 | -0.20 | 0.11 | -1.87 | 0.06 | -0.42 | 0.01 |
| PXA (*n*=2) | 10.4 | -0.17 | 0.07 | -2.42 | 0.02 | -0.31 | -0.03 |
| PXA (*n*=2) | 22.5 | -0.13 | 0.10 | -1.35 | 0.18 | -0.32 | 0.06 |

**Supplementary Table 2a Detailed moderator analysis for the effect of the pathology type on the relationship between spike rates and distance at different lesion volumes**

| Pathology Type | *At Lesion Volume (cm^3^)* | *Effect* | *HCSE* | *t* | *p* | *LLCI* | *ULCI* |
| --- | --- | --- | --- | --- | --- | --- | --- |
| Cavernoma (*n*=2) | 0.5 | -1.02 | 2.65 | -0.39 | 0.70 | -6.22 | 4.17 |
| Cavernoma (*n*=2) | 10.4 | -1.12 | 2.71 | -0.41 | 0.68 | -6.43 | 4.19 |
| Cavernoma (*n*=2) | 22.5 | -1.24 | 3.01 | -0.41 | 0.68 | -7.15 | 4.66 |
| DNET (*n*=5) | 0.5 | 0.43 | 2.02 | 0.21 | 0.83 | -3.53 | 4.40 |
| DNET (*n*=5) | 10.4 | 0.33 | 1.38 | 0.24 | 0.81 | -2.38 | 3.04 |
| DNET (*n*=5) | 22.5 | 0.21 | 0.73 | 0.29 | 0.77 | -1.23 | 1.65 |
| FCD (*n*=12) | 0.5 | -0.32 | 0.24 | -1.37 | 0.17 | -0.78 | 0.14 |
| FCD (*n*=12) | 10.4 | -0.42 | 0.47 | -0.90 | 0.37 | -1.34 | 0.50 |
| FCD (*n*=12) | 22.5 | -0.55 | 1.32 | -0.41 | 0.68 | -3.14 | 2.05 |
| GG (*n*=9) | 0.5 | 0.06 | 0.50 | 0.12 | 0.91 | -0.92 | 1.03 |
| GG (*n*=9) | 10.4 | -0.04 | 0.26 | -0.16 | 0.88 | -0.56 | 0.48 |
| GG (*n*=9) | 22.5 | -0.17 | 1.08 | -0.15 | 0.88 | -2.29 | 1.96 |
| LG Glioma (*n*=3) | **0.5** | **2.28** | **0.56** | **4.05** | **<0.0001** | **1.17** | **3.38** |
| LG Glioma (*n*=3) | **10.4** | **2.18** | **0.46** | **4.74** | **0.0001** | **1.28** | **3.08** |
| LG Glioma (*n*=3) | 22.5 | 2.06 | 1.18 | 1.73 | 0.08 | -0.27 | 4.38 |
| PXA (*n*=2) | 0.5 | 0.26 | 1.01 | 0.26 | 0.80 | -1.73 | 2.25 |
| PXA (*n*=2) | 10.4 | 0.16 | 0.59 | 0.27 | 0.78 | -0.99 | 1.31 |
| PXA (*n*=2) | 22.5 | 0.04 | 0.90 | 0.04 | 0.97 | -1.74 | 1.81 |

**Supplementary Table 2b Detailed moderator analysis for the effect of the pathology type on the relationship between ripple rates and distance at different lesion volumes**

Moderator analysis of the influence of *Pathology Type* (categorical) at small, moderate and large *Lesion Volumes* (continuous) on the relationship between *spike*/*ripple* *rate* and *distance*. A negative value for *Effect* indicates the pathology type moderates the relationship between spike/ripple rate and distance towards a more negative slope at the specific lesion volume. A positive value indicates the opposite. Statistically significant moderation by a pathology type at a certain lesion volume is highlighted in bold.

*Lesion Volume* alone did not show statistically significant moderation effects. Neither *Pathology Type* nor *Lesion Volume* showed a significant moderation effect on the relationship between *fast ripple rate* and *distance*.

Abbreviations: dysembryoplastic neuroepithelial tumor (DNET), focal cortical dysplasia (FCD), ganglioglioma (GG), Low-grade glioma (LG Glioma), pleomorphic xanthoastrocytoma (PXA), coefficient *B*, heteroscedasticity consistent standard error (*HCSE*), Lower Limit Confidence Interval (LLCI), Upper Limit Confidence Interval (ULCI)*.*
